# Pyocin S5 Import into Pseudomonas aeruginosa Reveals a Generic Mode of Bacteriocin Transport

**DOI:** 10.1128/mBio.03230-19

**Published:** 2020-03-10

**Authors:** Hannah M. Behrens, Edward D. Lowe, Joseph Gault, Nicholas G. Housden, Renata Kaminska, T. Moritz Weber, Catriona M. A. Thompson, Gaëtan L. A. Mislin, Isabelle J. Schalk, Daniel Walker, Carol V. Robinson, Colin Kleanthous

**Affiliations:** a Department of Biochemistry, University of Oxford, Oxford, United Kingdom; b Chemistry Research Laboratory, University of Oxford, Oxford, United Kingdom; c Institute of Bioorganic Chemistry, Heinrich Heine University Düsseldorf, Forschungszentrum Jülich, Jülich, Germany; d Institute of Infection, Immunity, and Inflammation, College of Medical, Veterinary, and Life Sciences, University of Glasgow, Glasgow, United Kingdom; e UMR 7242, Biotechnologie et Signalisation Cellulaire, ESBS, Illkirch, France; Institut Pasteur

**Keywords:** membrane, pyocin, transport

## Abstract

Pyocin S5 (PyoS5) is a potent protein bacteriocin that eradicates the human pathogen Pseudomonas aeruginosa in animal infection models, but its import mechanism is poorly understood. Here, using crystallography, biophysical and biochemical analyses, and live-cell imaging, we define the entry process of PyoS5 and reveal links to the transport mechanisms of other bacteriocins. In addition to its C-terminal pore-forming domain, elongated PyoS5 comprises two novel tandemly repeated kinked 3-helix bundle domains that structure-based alignments identify as key import domains in other pyocins. The central domain binds the lipid-bound common polysaccharide antigen, allowing the pyocin to accumulate on the cell surface. The N-terminal domain binds the ferric pyochelin transporter FptA while its associated disordered region binds the inner membrane protein TonB1, which together drive import of the bacteriocin across the outer membrane. Finally, we identify the minimal requirements for sensitizing Escherichia coli toward PyoS5, as well as other pyocins, and suggest that a generic pathway likely underpins the import of all TonB-dependent bacteriocins across the outer membrane of Gram-negative bacteria.

## Abstract (precis)

Bacteriocins are toxic polypeptides made by bacteria to kill their competitors, making them interesting as potential antibiotics. Here, we reveal unsuspected commonalities in bacteriocin uptake pathways, through molecular and cellular dissection of the import pathway for the pore-forming bacteriocin pyocin S5 (PyoS5), which targets Pseudomonas aeruginosa. In addition to its C-terminal pore-forming domain, PyoS5 is composed of two tandemly repeated helical domains that we also identify in other pyocins. Functional analyses demonstrate that they have distinct roles in the import process. One recognizes conserved sugars projected from the surface, while the other recognizes a specific outer membrane siderophore transporter, FptA, in the case of PyoS5. Through engineering of Escherichia coli cells, we show that pyocins can be readily repurposed to kill other species. This suggests basic ground rules for the outer membrane translocation step that likely apply to many bacteriocins targeting Gram-negative bacteria.

## INTRODUCTION

Bacteria living within communities do so through cooperation and antagonism. Forms of antagonism whereby one bacterium targets another are important for maintaining the stable coexistence of bacteria within microbiomes and are deployed by pathogens and commensals alike to kill competitors (1). Antagonism occurs via several routes, the most common being bacteriocins, contact-dependent inhibition, or type VI secretion. Of these, only the release of bacteriocins does not rely on physical contact between bacterial cells. Bacteriocin production generally occurs following a stress signal, such as DNA damage, inducing expression and release of the bacteriocin from autolysed cells (2). The bacteriocin then diffuses through the medium to kill a neighboring cell. Bacteriocins range in size from small peptides to large proteins, with both types currently being evaluated/developed as antimicrobials against multidrug-resistant bacteria (3, 4). In many instances, however, developments are hindered by a lack of understanding as to how these molecules work. In the case of protein bacteriocins, extensive sequence diversification and homologous recombination further hamper efforts to find generic mechanisms of uptake. Here, we focus on the uptake mechanism of PyoS5, a protein bacteriocin that specifically targets the opportunistic human pathogen Pseudomonas aeruginosa and that was shown recently in animal models to be more effective at clearing lung infections than was tobramycin, the antibiotic generally used to treat P. aeruginosa in cystic fibrosis patients (5). Through a structure-led approach, we deconstruct the energized uptake pathway of PyoS5 and show that its transport across the outer membrane likely represents the default pathway for all TonB-dependent bacteriocins.

There is a pressing need for new antibiotics against Gram-negative bacteria, in particular for P. aeruginosa, which has been designated a priority pathogen (6). The intrinsic low permeability of its outer membrane renders P. aeruginosa insensitive to many classes of antibiotics. Many strains also express multiple drug efflux pumps and carbapenemases, making P. aeruginosa one of the major causes of nosocomial infections in the developed and developing world. One class of molecule that readily translocates across the impervious outer membrane of P. aeruginosa to deliver a cytotoxin is the S-type pyocins, which are 40- to 90-kDa protein bacteriocins made by P. aeruginosa. Indeed, a recent survey showed that >85% of P. aeruginosa strains encode nuclease-type pyocins within their genomes (7), hinting at the importance of these protein antibiotics to interstrain competition.

S-type pyocins (here, pyocins) belong to a broad group of protein bacteriocins that includes colicins which kill Escherichia coli, as well as bacteriocins that target other Gram-negative bacteria, such as Klebsiella pneumoniae, Serratia marcescens, and Yersinia pestis. Colicins, like pyocins, exploit the proton motive force (PMF) to translocate through the cell envelope to deliver a cytotoxic domain, typically a pore-forming domain or a nuclease that cleaves DNA, rRNA, or tRNA (8). Also like colicins, pyocins are multidomain toxins, and their constituent domains are associated with binding outer membrane receptors and the import process itself. There are currently several structures for intact colicins in the Protein Data Bank (PDB) but only two for pyocins, PaeM and L1 (9, 10). However, PaeM and L1 are atypical among the bacteriocins due to their small sizes (14 kDa for PaeM and 28 kDa for L1 compared to >50 kDa for most pyocins). Consequently, we know very little about the structural biology of typical pyocins found in P. aeruginosa genomes. Structural data are important to understanding bacteriocin uptake mechanisms, especially since the domain arrangement of pyocins is different from that of colicins. The receptor-binding domains are centrally located in colicins, and their membrane translocation domains are at the N terminus, whereas in pyocins, the order is reported to be reversed (11). This change in relative domain orientation would mean a fundamental difference in how these molecules are transported across the outer membrane.

PyoS5 delivers a pore-forming domain across the outer membrane to depolarize the cell, while PyoS5-producing cells are protected against the action of the toxin by ImS5, a small membrane-localized immunity protein (12). Previous work has shown that PyoS5 binds the lipopolysaccharide (LPS)-anchored common polysaccharide antigen (CPA), which is identical across P. aeruginosa strains (13) and is a major surface antigen in cystic fibrosis isolates (14), and that PyoS5 susceptibility depends on the ferric pyochelin transporter FptA (15). Here, we delineate how PyoS5, by parasitizing FptA and CPA in the outer membrane and in conjunction with proton motive force (PMF)-linked TonB1 in the inner membrane, delivers its cytotoxic domain into P. aeruginosa. By defining the functionality of all constituent domains of PyoS5, we show that pyocins and colicins are similarly constructed with respect to the relative positions of the receptor-binding and translocation domains, a conclusion reinforced by the demonstration that PyoS5 can kill suitably engineered E. coli strains.

## RESULTS AND DISCUSSION

### The structure of PyoS5 reveals a novel domain architecture.

**FIG 1:**
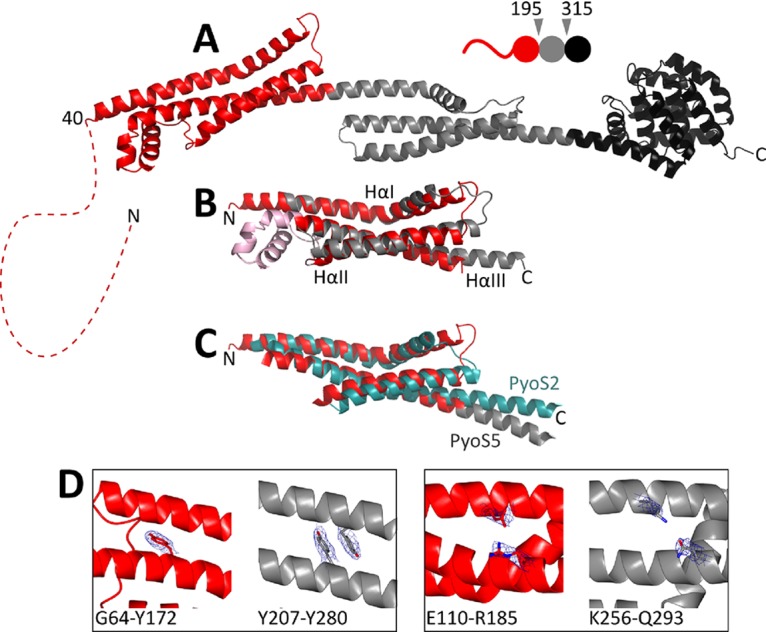
Crystal structure of PyoS5. (A) The 2.2-Å crystal structure of PyoS5 (residues 40 to 505). The first kTHB domain is in red (residues 40-196), the second kTHB is in gray (residues 195-315), and the pore-forming domain is in black (residues 316-505). Residues 2 to 39 are not resolved and are represented (to scale) by a red dashed line. (B) Structural alignment of PyoS5_40–196_ (red) and PyoS5_194–315_ (gray), with an RMSD of 2.5 Å. Residues 123 to 162 (pink) are not conserved in PyoS5_194–315_ and were excluded from the alignment. (C) Structural alignment of PyoS5 kTHB domains (red and gray) with that from PyoS2 (teal), with an RMSD of 4.1 Å. PyoS5 residues 40 to 213 are shown, with residues 123 to 156 excluded, and PyoS2 residues 46 to 206 are shown, with residues 124 to 151 excluded. (D) Interactions within domain 1 (red) and domain 2 (gray) are not conserved, as illustrated by the exemplary interactions shown. Electron density is shown, with a cutoff of 1 σ.

PyoS5 was expressed and purified from E. coli cells (see Materials and Methods). The 57-kDa toxin was monomeric in solution and active against P. aeruginosa strains at subnanomolar concentrations (see Fig. S1 in the supplemental material). The protein crystallized in the P2_1_ space group, and the structure was solved by a combination of single-wavelength anomalous diffraction and molecular replacement to a resolution of 2.2 Å (Fig. 1A and Table S1) (see Materials and Methods). The first 39 residues were absent from the final model and presumed to be unstructured; we refer to this below as the “disordered region.” Otherwise, continuous electron density was observed for the entirety of the remaining protein sequence (residues 40 to 498). The structure shows that PyoS5 is an elongated, α-helical protein measuring 36 Å on the short axis and 195 Å on the long axis. Colicins are similarly long proteins and have disordered N termini (16–18). The extended conformation was confirmed by small-angle X-ray scattering (SAXS) data; 93% of the modeled PyoS5 residues were within the SAXS envelope (Fig. S2A and B). Also similar to colicins is the prevalence of an α-helical structure in PyoS5. PyoS5 contains 17 helices, the high preponderance of helical structure likely reflecting the need to forcibly unfold the toxin during transport into a cell and the lower forces known to be required for unfolding helices relative to β-sheets (reviewed in reference 19).

10.1128/mBio.03230-19.1 FIG S1 Characterization of purified PyoS5. (A) Coomassie-stained SDS-PAGE showing purified PyoS5, compared to molecular weight standards (Fermentas unstained protein marker). (B) Electrospray ionization mass spectrometry (ESI-MS) of full-length PyoS5 gives a mass of 57,008.00 Da, compared with a sequence-based expected mass of 58,007.98 Da. (C) PyoS5 spotted on P. aeruginosa YHP17 in three-fold dilutions starting at 10 μM. Zones of clearance indicate growth inhibition by PyoS5. (D) Size exclusion multiangle light scattering (SEC-MALS) of PyoS5 at 9.5 mg/ml (black), 4.8 mg/ml (gray), 2.4 mg/ml (red), 1.1 mg/ml (blue), and 0.6 mg/ml (yellow) shows PyoS5 is monomeric at all tested concentrations. Download FIG S1, PDF file, 0.2 MB. Copyright © 2020 Behrens et al. 2020 Behrens et al. This content is distributed under the terms of the Creative Commons Attribution 4.0 International license.

10.1128/mBio.03230-19.2 FIG S2 SAXS data of PyoS5. (A) SAXS of PyoS5 (black) at 5.4 mg/ml shows that PyoS5 is monodispersed. The theoretical scattering curve for the crystal structure (red line) fits the experimental SAXS data well, with a χ^2^ of 1.74. (B) Fit of the PyoS5 crystal structure into the PyoS5 SAXS envelope by Chimera shows 93% of atoms within the SAXS envelope. (C) Comparison of pore-forming domains from bacteriocins. All available structures of pore-forming domains were aligned and colored in a gradient from yellow (N terminus) to red (C terminus). The pores are shown in order of decreasing alignment quality (Q) score. All pores in the left column have immunity proteins that pass the membrane three times, and those in the right column have immunity proteins that pass the membrane four times. RMSDs, Q scores, and immunity protein topologies are shown. The following residues were aligned to PyoS5315 to 498: ColIa448 to 624 (PDB 1CII), ColE1345 to 522 (PDB 2I88), ColN188 to 486 (PDB 1A87), ColA393 to 591 (PDB 1COL), ColS4299 to 499 (PDB 3FEW), and ColB312 to 511 (PDB 1RH1). (D) Phylogenetic tree of kTHB domains with branch lengths in substitutions per site (gray) and percent protein sequence identity to the most similar crystalized kTHB (blue and red). (E) Sequence alignment of the three kTHBs that have been shown to bind CPA. Identical residues are shown in green. Download FIG S2, PDF file, 1.1 MB. Copyright © 2020 Behrens et al. 2020 Behrens et al. This content is distributed under the terms of the Creative Commons Attribution 4.0 International license.

10.1128/mBio.03230-19.9 TABLE S1 X-ray data processing, refinement, and validation statistics for PyoS5. Download Table S1, DOCX file, 0.03 MB. Copyright © 2020 Behrens et al. 2020 Behrens et al. This content is distributed under the terms of the Creative Commons Attribution 4.0 International license.

The structure of PyoS5 is composed of three ordered domains (Fig. 1A). The C-terminal domain (domain 3, residues 315 to 498) has the canonical 10-helix bundle fold of a pore-forming domain found in colicins (20), which is consistent with the killing activity of PyoS5 (12). Previous studies have highlighted that the protective immunity proteins of pore-forming domains within colicins fall into two subgroups, although the functional significance of this is unclear. Immunity proteins against colicins A, B, and N (the so-called A type) have four transmembrane helices, while those against colicins E1, Ia, and K (the so-called E1 type) have three transmembrane helices (20). Based on the predicted number of transmembrane helices of its immunity protein, the pore-forming domain of PyoS5 belongs to the E1 type (21). Through detailed structural comparisons of all pore-former domains with that of PyoS5, we identified a clear structural difference between the pore-forming domains of the A and E1 groups (Fig. S2C). Specifically, this difference relates to the positioning of helices 1 and 5 of the domain with respect to each other; in A-type structures, helix 1 is positioned close to the center of the domain, pushing out helix 5, while in E1-type structures, helix 5 is located closer to the center of the domain. These pore-forming domain structures represent the ground state of the ionophore before depolarization of the inner membrane. We speculate that the structural alterations evident in the A and E1 groups may reflect differences in the way each class of pore-forming domain is recognized by its particular type of immunity protein before insertion in the bacterial inner membrane.

The other structured domains of PyoS5 are also helical bundles but of a novel fold. Domain 1 comprises residues 40 to 194, while domain 2 comprises residues 195 to 315. The core structural motif of each domain is a kinked three-helix bundle (kTHB). The two kTHB domains are structurally similar to each other (superposition root mean square deviation [RMSD], 2.5 Å) but share little sequence identity (∼12%) (Fig. 1B). Each kTHB domain is composed of a kinked helix I connected to a straight helix II by a loop. Helix II packs against both helix I and a third straight helix, helix III. The connection between helices II and III varies between the two copies of the fold. In domain 1, this connection is composed of three short helical turns, while in domain 2, it is a loop. The other striking feature of the kTHB structural motif is that the third helix from each domain extends into the next domain of the pyocin; helix III of domain 1 extends over 90 Å into domain 2, where it forms helix I, while helix III of domain 2 extends over 90 Å to the pore-forming domain of the toxin. The kTHB fold is stabilized predominantly by hydrophobic interactions mediated by aliphatic amino acid side chains and, in one instance, aromatic stacking (Tyr207 to Tyr280, domain 2) (Fig. 1D). None of these stabilizing interactions are conserved.

Recently, White et al. reported the structure of the N-terminal domain of the nuclease pyocin PyoS2 bound to the outer membrane protein FpvAI (22). We found by structural superposition that the kTHB domain 1 of PyoS5 is structurally similar to this domain of PyoS2 (Fig. 1C), and the sequence similarity of 75% between the second domains of PyoS5 and PyoS2 suggests similar structures here as well (Fig. S2D and E). Sequence similarities of domains in pyocins S1, SD1, SD2, S3, SD3, and S4 to the kTHB domain also suggest these are common among pyocins (Fig. S2D). The structural superposition of the PyoS5 and PyoS2 kTHB domains, without the small helices connecting helix II and helix III in PyoS5, has an RMSD of 4.1 Å over 128 residues (Fig. 1C).

We conclude that PyoS5 is an elongated bacteriocin comprising a disordered region at its N terminus, two kTHB domains, which is a common structural platform for protein bacteriocins targeting P. aeruginosa, and a C-terminal pore-forming domain. We next set out to ascribe functions to each of the domains/regions of PyoS5 that transport the pore-forming domain into P. aeruginosa cells.

### Functional annotation of PyoS5 domains.

We expressed and purified truncations of PyoS5 that removed one or more domains/regions. These included PyoS5_1–315_, in which the pore-forming domain was removed, PyoS5_1–196_, in which both domain 2 and the pore-forming domain were deleted, and PyoS5_194–315_, which only contained domain 2. The constructs were folded, as determined by circular dichroism spectroscopy, and their thermal melting temperatures largely recapitulated those found in intact PyoS5 (Fig. S3).

10.1128/mBio.03230-19.3 FIG S3 Circular dichroism (CD) spectra of PyoS5 constructs. (A) CD spectra of 0.1 mg/ml PyoS5_1–315_ (black), PyoS5_1–196_ (red), and PyoS5_194–315_ (gray) at RT in 20 mM NaCl and 10 mM potassium phosphate buffer (pH 7.5) show that the PyoS5 domains are helical and folded. (B) CD thermal melts of 0.1 mg/ml PyoS5_1–315_ (black), with a melting temperature (*T_m_*) of 51.0 ± 0.1°C, PyoS5_1–196_ (red), with a *T_m_* of 57.5 ± 0.0°C, and PyoS5_194–315_ (gray), with a *T_m_* of 43.7 ± 0.3°C in 20 mM NaCl and 10 mM potassium phosphate buffer (pH 7.5). Two repeats were performed; one is shown as circles with its fit as a line. Download FIG S3, PDF file, 0.05 MB. Copyright © 2020 Behrens et al. 2020 Behrens et al. This content is distributed under the terms of the Creative Commons Attribution 4.0 International license.

**FIG 2:**
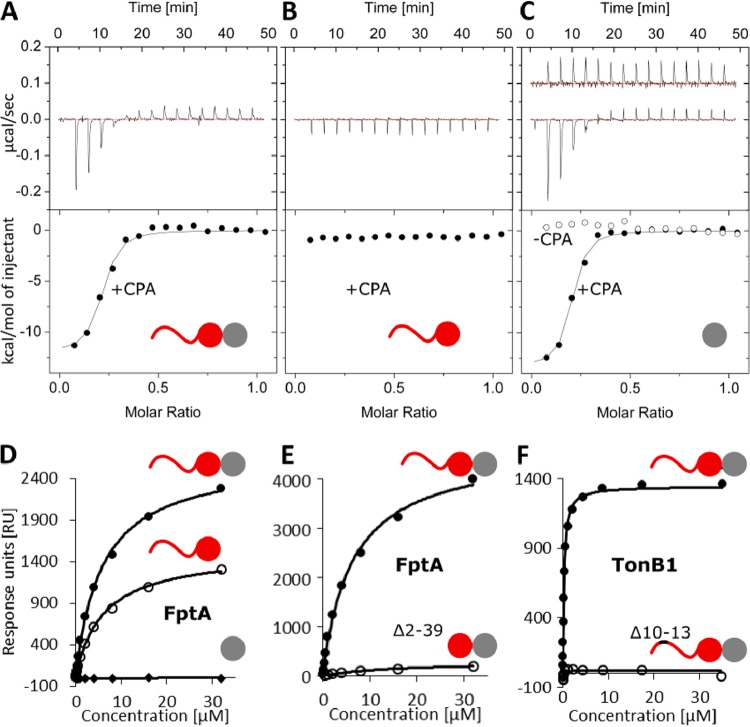
kTHB domain 2 binds CPA, kTHB domain 1 binds FptA, and the N-terminal disordered region binds TonB1. (A and B) ITC data for PyoS5_1–315_ titrated into P. aeruginosa PAO1 LPS-derived polysaccharide containing CPA and OSA (closed circles) gives a *K_d_* of 612 ± 332 nM (A), and PyoS5_1–196_ titrated into P. aeruginosa PAO1 LPS-derived polysaccharide shows no binding (B). (C) ITC data for PyoS5_194–315_ titrated into P. aeruginosa PAO1 LPS-derived polysaccharide gives a *K_d_* of 269 ± 44 nM. PyoS5_194–315_ titrated into P. aeruginosa Δ*rmd* LPS-derived polysaccharide containing OSA only (open circles) shows no binding. (A to C) *K_d_* values and concentrations can be found in Table S2B. All ITC experiments were performed in duplicate in 0.2 M Na-phosphate buffer (pH 7.5) at 25°C, and one repeat is shown. Data were corrected for heats of dilution by subtracting the average of the last five injections and fit to a model of single-site binding. (D) SPR data for FptA (0.03 to 32 μM) binding to PyoS5_1–315_ (closed circles; *K_d_*, 6.5 ± 0.4 μM), PyoS5_1–196_ (open circles; *K_d_*, 7.1 ± 0.7 μM), or PyoS5_194–315_ (diamonds; no binding). PyoS5_1–315_ achieves higher RU levels than does PyoS5_1–196_ even though the binding affinity remains essentially unchanged. Since the pyocin was immobilized randomly on the chip, this is likely due to the greater availability of FptA binding sites in the larger (PyoS5_1–315_) construct. (E) SPR data for FptA (0.03 to 32 μM) binding to PyoS5_1–315_ (closed circles; *K_d_*, 6.5 ± 0.4 μM) or PyoS5_1–315_ Δ2–39 (open circles; *K_d_*, 14.7 ± 0.4 μM). (F) SPR data for TonB1 (0.009 to 35 μM) binding to PyoS5_1–315_ (closed circles; *K_d_*, 241 ± 9 nM) or PyoS5_1–315_ Δ10–13 (open circles; no binding). (D to F) One of three repeats is shown. All experiments were performed in parallel on the same chip in HBS-OG buffer at 25°C. All ligands were immobilized by amine coupling, and sensorgram data were extracted and fit with a 1:1 binding model. *K_d_* values are presented in Table S2C.

We first analyzed the capacity of PyoS5 and the various deletion constructs to bind CPA in isothermal titration calorimetry (ITC) experiments. Heats of binding were observed for PyoS5_1–315_ and PyoS5_194–315_ but not PyoS5_1–196_ (in 0.2 M Na-phosphate buffer [pH 7.5]) (Fig. 2A to C and Table S2A). From these experiments, equilibrium dissociation constant (*K_d_*) values of 0.6 μM for PyoS5_1–315_ and 0.3 μM for PyoS5_194–315_ were obtained, similar to that reported previously for intact PyoS5 binding CPA (13). When polysaccharides derived from P. aeruginosa PAO1 Δ*rmd* were used (that do not contain CPA), no binding to PyoS5_194–315_ was detected (Fig. 2C). These results demonstrate that the CPA-binding activity of PyoS5 resides within domain 2 and that the CPA-binding function is not a conserved feature of the kTHB fold. Pyocins S2 and SD3 have also been shown previously to bind P. aeruginosa CPA sugars (13). Sequence alignments show that each has a domain equivalent to that of domain 2 of PyoS5. Indeed, the level of sequence identity across this region (39%) is far greater than that between the two kTHB domains of PyoS5. Moreover, over half of the 45 identical residues shared between pyocins S2, SD3, and S5 form a grooved surface that runs perpendicular to the long axis of PyoS5 (Fig. S2E and S4). We infer that this conserved groove is the CPA-binding site in these different pyocins, each of which nevertheless delivers a different cytotoxic domain into P. aeruginosa.

10.1128/mBio.03230-19.4 FIG S4 Conserved residues between PyoS2, PyoSD3, and PyoS5 CPA-binding domains. Residues identical in all three CPA-binding domains (red) are mapped onto PyoS5 CPA-binding kTHB domain 2 (gray). Surface and cartoon representations are shown at two different angles. Download FIG S4, PDF file, 0.4 MB. Copyright © 2020 Behrens et al. 2020 Behrens et al. This content is distributed under the terms of the Creative Commons Attribution 4.0 International license.

10.1128/mBio.03230-19.10 TABLE S2 Interactions of PyoS5 with cell envelope components. Download Table S2, DOCX file, 0.03 MB. Copyright © 2020 Behrens et al. 2020 Behrens et al. This content is distributed under the terms of the Creative Commons Attribution 4.0 International license.

PyoS5-mediated killing of P. aeruginosa cells requires the ferric pyochelin transporter FptA, and the central region of the toxin (residues 151 to 300) has been implicated in defining this specificity (15). This region corresponds largely to domain 2 in the PyoS5 crystal structure, which, as the work described above indicates, is involved in CPA binding. We therefore investigated PyoS5 binding to FptA and identified the region involved. Initially, we used native mass spectrometry (MS) to verify that PyoS5 binds FptA (Table S2B). We then determined the affinity for the complex using surface plasmon resonance (SPR) where the pyocin and various deletion constructs were immobilized on the chip (Fig. 2D and Table S2C). These experiments determined the *K_d_* for the PyoS5-FptA complex to be 6.5 μM (in 25 mM HEPES buffer [pH 7.5], 150 mM NaCl, 1% [wt/vol] *n*-octyl-β-d-glucoside [β-OG]). Upon the addition of ferric pyochelin to our SPR experiments, the binding of PyoS5 to FptA reduced significantly (Fig. S5B), suggesting that the binding sites for the pyocin and pyochelin overlap. This result was confirmed by native-state MS experiments where PyoS5 dissociated preformed complexes of ferric pyochelin bound to FptA (Fig. S5A). We next delineated the FptA binding site in PyoS5. The deletion of domain 2 had a marginal effect on FptA binding, while domain 2 alone showed no FptA binding (Fig. 2D and Table S2C). Deletion of the disordered region at the N terminus of PyoS5 (residues 2 to 39) had a large effect on the amount of FptA that could bind to the chip (Fig. 2E), suggesting that this was affecting binding. However, closer examination indicated that binding was affected only 2-fold (Table S2C) and that the impact of the truncation was likely due to restricted access of FptA to its binding site on domain 1 in this construct (Fig. 2E and Table S2C). In contrast, when the first 12 residues of the mature construct were deleted (Δ2–9 and Δ10–13), binding to FptA remained unaffected (Table S2C). We conclude that the FptA binding site in PyoS5 is predominantly localized to kTHB domain 1 with a minor contribution from its associated disordered region at the N terminus.

10.1128/mBio.03230-19.5 FIG S5 Pyochelin and PyoS5 compete for FptA binding. (A) FptA was incubated with 4-fold excess ferric pyochelin (Pch) for 45 min, and then excess Pch was removed by desalting. The native mass spectrum of this FptA sample (19 μM) in the presence of 19 μM PyoS5_1–299_ shows species with masses consistent with free FptA (gray), FptA-Pch complex (red), FptA-PyoS5_1–299_ complex (blue), and dissociated FptA (yellow). No complex of FptA, PyoS5_1–299_, and Pch is detected. (B) SPR shows 3.4 μM FptA binding to immobilized PyoS5_1–315_ in the presence of premixed Pch and FeCl_3_ at ratios of 1:0:0 (black), 1:2:8 (gray), and 1:10:40 (red) FptA:Pch:FeCl_3,_ respectively. An excess of iron over Pch was chosen to ensure a high percentage of ferric Pch. With a 10-fold excess of Pch, no binding of FptA to PyoS5_1–315_ is observed. Download FIG S5, PDF file, 0.07 MB. Copyright © 2020 Behrens et al. 2020 Behrens et al. This content is distributed under the terms of the Creative Commons Attribution 4.0 International license.

All protein bacteriocins access the PMF via either the Tol or Ton system of Gram-negative bacteria (generally referred to as group A and B toxins in the colicin literature, respectively), which they use to drive translocation across the outer membrane (2). It has yet to be established which of these systems is contacted by PyoS5. Typically, Tol/Ton dependence is evaluated using deletion strains. We focused initially on Ton dependence since deletion strains in P. aeruginosa PAO6609 are available (Tol is essential in P. aeruginosa). P. aeruginosa harbors three *tonB* genes, *tonB1*, *tonB2*, and *tonB3* (23–25). PAO6609 is a derivative of P. aeruginosa PAO1 and so is naturally immune to PyoS5 because it harbors the ImS5 immunity gene (26). We therefore generated a PyoS5-ColIa chimera in which the pore-forming domain of PyoS5 was substituted for that of colicin Ia to overcome this immunity. PyoS5-ColIa was active against P. aeruginosa PAO6609 and strains with *tonB2* and *tonB3* deleted (Fig. S6A). It was not possible to test the susceptibility of a *tonB1* deletion strain because the high levels of iron needed for the growth of this strain diminished PyoS5-ColIa chimera susceptibility in the parent P. aeruginosa PAO6609, most likely due to iron-dependent downregulation of FptA expression (27). We therefore resorted to direct SPR binding assays to determine if PyoS5 bound purified TonB1 *in vitro* (see Materials and Methods for further details). We found that TonB1 binds PyoS5_1–315_ with an affinity of 230 nM in SPR experiments (Fig. 2F and Table S2C). Moreover, a putative 9-residue TonB box, found in TonB1-dependent transporters and bacteriocins utilizing TonB1, is also found in the N-terminal disordered region of PyoS5 (residues 6 to 14). The deletion of residues 10 to 13 abolished binding to TonB1, confirming this region to be the TonB1 binding site (Fig. 2F and Table S2C).

10.1128/mBio.03230-19.6 FIG S6 PyoS5-ColIa is not TonB2 or TonB3 but CPA dependent. (A) Zones of clearance from PyoS5-ColIa did not differ between parent strain PA6609, TonB2-deficient strain K1408, TonB3-deficient strain MS231, and TonB2- and TonB3-deficient strain MS233. A 3-fold dilution series starting at 10 μM PyoS5-ColIa was added to strains grown on LB agar. (B) CPA increases susceptibility to PyoS5-ColIa. A 3-fold serial dilution starting at 10 μM spotted onto the soft agar containing P. aeruginosa PAO1 or P. aeruginosa PAO1 Δ*rmd* from top left to bottom right. In the CPA-deficient Δ*rmd* mutant strain, a 9-fold reduction in susceptibility but not complete resistance is observed. Download FIG S6, PDF file, 0.3 MB. Copyright © 2020 Behrens et al. 2020 Behrens et al. This content is distributed under the terms of the Creative Commons Attribution 4.0 International license.

In summary, through a combination of biophysical and structural approaches, we have delineated the major binding interactions of PyoS5 with the P. aeruginosa cell envelope. Of the two kTHB domains, domain 2 binds CPA, while domain 1 binds the ferric pyochelin transporter FptA, with a minor contribution by the disordered region, which in addition binds the inner membrane protein TonB1.

### Surface accumulation and energized import of fluorescently labeled PyoS5 into P. aeruginosa PAO1 cells.

**FIG 3:**
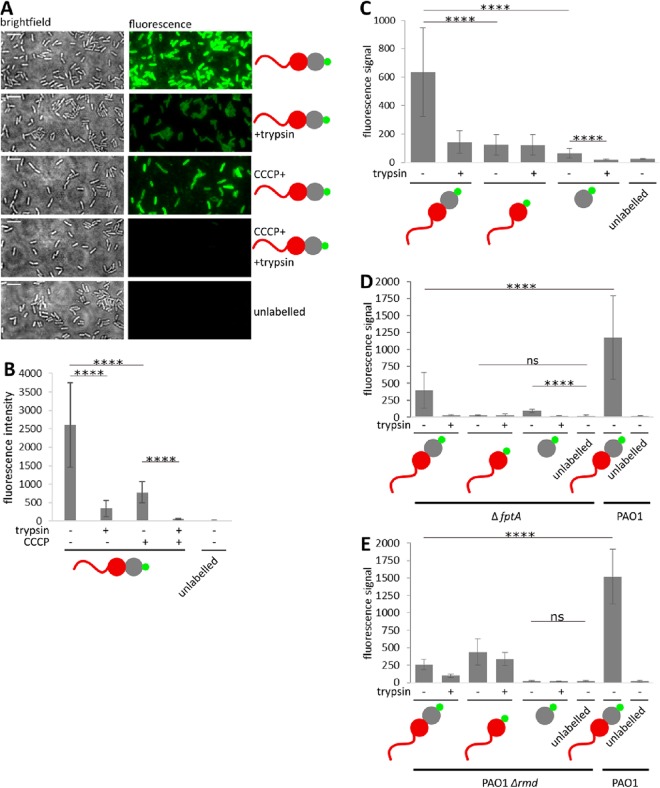
CPA accumulates PyoS5 at the cell surface while FptA and TonB1 mediate import. (A) Fluorescent labeling of live P. aeruginosa PAO1 cells with PyoS5_1–315_-AF^488^. Additionally, the effects of depleting the PMF with CCCP before incubation with PyoS5_1–315_-AF^488^ and of trypsin treatment to remove surface-exposed PyoS5_1–315_-AF^488^ after incubation with PyoS5_1–315_-AF^488^ were examined. Scale bars = 5 μm. (B) Quantification of the average cell fluorescence observed under different conditions tested in panel A. (C) Fluorescent labeling of live P. aeruginosa PAO1 using PyoS5_1–315_-AF^488^, PyoS5_1–196_-AF^488^, and PyoS5_194–315_-AF^488^ with and without trypsin treatment quantified to determine the average cell fluorescence. (D) Fluorescent labeling of live P. aeruginosa PW8161 (Δ*fptA* mutant) and P. aeruginosa PAO1 using PyoS5_1–315_-AF^488^, PyoS5_1–196_-AF^488^, and PyoS5_194–315_-AF^488^ with and without trypsin treatment was quantified to determine the average cell fluorescence. (E) Fluorescent labeling of live P. aeruginosa PAO1 Δ*rmd* and PAO1 using PyoS5_1–315_-AF^488^, PyoS5_1–196_-AF^488^, and PyoS5_194–315_-AF^488^ with or without trypsin treatment was quantified to determine the average cell fluorescence. (A to E) **** indicates a *P* value below 0.0001 in Student's *t* test; ns indicates nonsignificance.

We developed a fluorescence-based import assay for PyoS5 where the transport of all its domains, barring the pore-forming domain, could be visualized and where the energetics of import could be established. We replaced the pore-forming domain of PyoS5 with a C-terminal cysteine residue and labeled this residue with Alexa Fluor 488 (PyoS5_1–315_-AF^488^). P. aeruginosa PAO1 cells were used in these experiments since cytotoxic activity was not being monitored. PyoS5_1–315_-AF^488^ readily labeled P. aeruginosa PAO1 cells (Fig. 3A). Trypsin treatment of these labeled cells, to remove surface-bound PyoS5, reduced fluorescence intensity significantly (∼8-fold), but fluorescence was still associated with cells (Fig. 3A and B). Inclusion of the protonophore carbonyl cyanide *m*-chlorophenylhydrazone (CCCP) with the trypsin treatment completely eradicated this remaining fluorescence, suggesting that this protected fluorescence was internalized due to the PMF (Fig. 3A and B). We next generated AF^488^-labeled constructs where either domain 2 was removed (PyoS5_1–196_-AF^488^) or where only labeled domain 2 was added to cells (PyoS5_194–315_-AF^488^). Removal of the CPA-binding domain (domain 1, PyoS5_1–196_-AF^488^) decreased surface-bound fluorescence in the absence of trypsin, while the addition of trypsin still revealed internalized fluorescence (Fig. 3C). PyoS5_194–315_-AF^488^ (domain 2 construct), on the other hand, labeled cells much less efficiently (likely due to its weak binding of CPA on the surface), and all of this fluorescence was trypsin sensitive, suggesting no internalization (Fig. 3C).

Repeating these assays with P. aeruginosa PAO1 Δ*fptA* cells or using PyoS5_1–315_ Δ10–13-AF^488^, in which part of the TonB1 binding site (residues 10 to 13) was deleted, showed that trypsin-protected fluorophores (i.e., imported molecules) were no longer detected, consistent with PMF/TonB1-dependent import of PyoS5 across the outer membrane via FptA (Fig. 3D andS7). Finally, import assays were conducted using P. aeruginosa PAO1 Δ*rmd* cells, which lack CPA. Surface-associated fluorescence of PyoS5_1–315_-AF^488^ and susceptibility to PyoS5-ColIa were much reduced in these cells, consistent with CPA being required for surface accumulation of PyoS5, but imported fluorescence in a domain 2 deletion was unaffected (Fig. 3E and S6B).

10.1128/mBio.03230-19.7 FIG S7 Deletion of the PyoS5 TonB-box prevents translocation. (A) Fluorescent labelling of live P. aeruginosa PAO1 using PyoS5_1–315_-AF^488^ and PyoS5_1–315_ Δ10–13-AF^488^ with and without trypsin treatment. Scale bars = 5 μm. (B) Quantification of the average cell fluorescence observed under different conditions tested in panel A. **** indicates a *P* value below 0.0001 in Student’s *t* test, and ns indicates a *P* value above 0.05. Download FIG S7, PDF file, 0.2 MB. Copyright © 2020 Behrens et al. 2020 Behrens et al. This content is distributed under the terms of the Creative Commons Attribution 4.0 International license.

In summary, our fluorescence assays suggest that the import of PyoS5 occurs in two stages. Initial binding to CPA via the central kTHB domain leads to accumulation on the surface of P. aeruginosa. Thereafter, the first kTHB domain of the pyocin binds FptA in the outer membrane, which also likely acts as the translocation channel, allowing contact between the disordered TonB1 binding site of PyoS5 with TonB1 in the inner membrane and PMF-driven import of the toxin (model presented below).

### Engineering pyocin susceptibility in E. coli.

As with most bacteriocins, pyocins are specific for a subset of strains, in this case from P. aeruginosa, which reflects the array of cell envelope interactions required for import. Yet, common principles are beginning to emerge suggesting that generic import mechanisms may apply for all Gram-negative bacteria that exploit protein bacteriocins. We therefore devised a test of this hypothesis by engineering E. coli susceptibility toward PyoS5 utilizing our current understanding of its import pathway.

**FIG 4:**
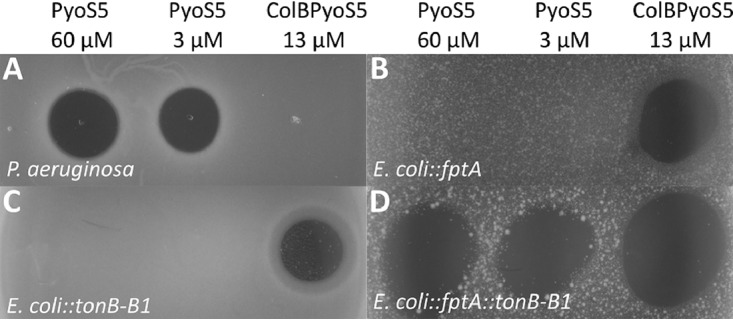
FptA and TonB1 constitute the minimal system for PyoS5 susceptibility in E. coli. (A to D) Susceptibility to PyoS5 (3 and 60 μM) was assessed for P. aeruginosa YHP17 (A), E. coli BL21(DE3) expressing FptA (B), E. coli BL21(DE3) expressing TonB-B1 (C), and E. coli BL21(DE3) expressing FptA and TonB-B1 (D). Zones of clearance are observed in all E. coli samples for the ColB PyoS5 (13 μM) control (B to D) and for both concentrations of PyoS5 in E. coli expressing FptA and TonB-B1 (D). In the P. aeruginosa control, clearance zones were observed for PyoS5 (A).

Our strategy was based on first determining if the pore-forming domain of PyoS5, if imported, could kill E. coli cells and then engineering the minimal requirements into E. coli in order for PyoS5 to be recognized and transported. A similar strategy was reported by Bosák et al., where E. coli was engineered to be susceptible to a bacteriocin specific for Yersinia kristensenii (28). In the present work, we first showed that a chimera of the PyoS5 pore-forming domain fused to the C terminus of the colicin B translocation and receptor-binding region (replacing colicin B’s own pore-forming domain) was cytotoxic against E. coli BL21(DE3) cells. We next challenged E. coli BL21(DE3) cells expressing P. aeruginosa FptA but saw no PyoS5 killing (Fig. 4). Rationalizing that E. coli TonB may not recognize the TonB1 binding sites (Ton boxes) of FptA and/or PyoS5, we also expressed a chimera of E. coli TonB (TonB_1–102_) fused to P. aeruginosa TonB1_201–342_ in E. coli BL21(DE3) cells expressing FptA. In this chimera, TonB-B1, the C-terminal domain, and periplasmic regions of TonB are those from P. aeruginosa, but the transmembrane domain that associates with TonB’s partner proteins ExbB and ExbD are those from E. coli. Under these conditions, E. coli became sensitized to PyoS5-mediated killing (Fig. 4). To determine the generality of this cross-species killing, we expressed the *fpvAI* gene, which is recognized by PyoS2 and PyoS4, in E. coli cells expressing the E. coli-P. aeruginosa TonB-B1 hybrid. This strain was sensitive to both PyoS2 and PyoS4 but not to PyoS5 (Fig. S8).

10.1128/mBio.03230-19.8 FIG S8 FpvAI and TonB1 constitute the minimal system for PyoS2 and PyoS4 susceptibility. (A to D) Susceptibility to 1 μM PyoS2, PyoS4, PyoS5, and ColB PyoS5 was assessed for P. aeruginosa YHP17 (A), E. coli BL21(DE3) expressing FpvAI (B), E. coli BL21(DE3) expressing TonB-B1 (C), and E. coli BL21(DE3) expressing FpvAI and TonB-B1 (D). Zones of clearance were observed for all E. coli conditions tested when ColB PyoS5 was applied (B to D). Similarly, E. coli expressing FpvAI and TonB-B1 was susceptible to both PyoS2 and PyoS4, as was the P. aeruginosa control (A and D). Zones of clearance for PyoS5 were only observed in P. aeruginosa. Download FIG S8, PDF file, 0.3 MB. Copyright © 2020 Behrens et al. 2020 Behrens et al. This content is distributed under the terms of the Creative Commons Attribution 4.0 International license.

We conclude that our engineered system is a simple means by which the import apparatus required for bacteriocins can be readily defined. Indeed, through this work, we discovered that PyoS4 is a TonB1-dependent bacteriocin. Importantly, our complete functional characterization of PyoS5 demonstrates that the prevailing view of receptor-binding and translocation domains being inverted in pyocins relative to colicins is not correct. Instead, pyocins and colicins are organized in the same way, which likely explains how a pyocin can be made to work in E. coli. They have central receptor-binding domains (kTHB domain 2 in PyoS5) and N-terminally located translocation domains (kTHB domain 1 and its associated disordered region). The confusion that has emerged in the field, that N-terminal domains of pyocins represent their receptor-binding domains, has arisen because pyocin interactions with their translocation channels (e.g., PyoS2 with FpvAI [22]) can be much higher affinity than the interaction of the pyocin with its initial CPA receptor. In summary, our results suggest that the underlying mechanisms by which Ton-dependent bacteriocins cross the outer membranes of the *Enterobacteriales* and *Pseudomonadales*, long thought to be unrelated, are fundamentally the same.

### Model for pyocin transport across the outer membrane of P. aeruginosa.

White et al. demonstrated recently that the N-terminal domain of PyoS2 translocates directly through FpvAI (22). The mechanism of import is analogous to that of FpvAI’s cognate siderophore ligand, ferripyoverdine; a labile portion of the transporter plug domain is removed by TonB1, allowing the TonB1 binding site (TonB box) of PyoS2 to enter the periplasm and activate import of the pyocin. Binding of PyoS2 to FpvAI is primarily through a short polyproline region that lacks a regular secondary structure and mimics pyoverdine. The principal binding site of PyoS5 for FptA is domain 1 and its associated disordered region, which does not, however, have an equivalent polyproline sequence. Its binding to FptA is also significantly weaker than that of PyoS2 for FpvAI. For both PyoS2 and PyoS5, however, the initial association with P. aeruginosa is by their central kTHB domains (domain 2 in PyoS5), which binds CPA embedded in the outer membrane and allows the toxin to decorate the cell surface (13).

**FIG 5:**
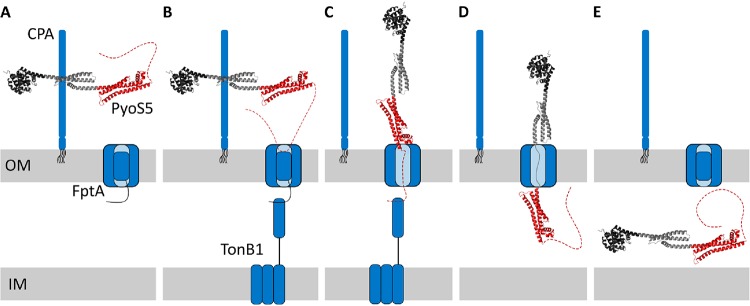
Model of PyoS5 import. (A) PyoS5 accumulates on the cell surface by binding to CPA through kTHB domain 2. (B) PyoS5 then contacts its outer membrane (OM) translocator, FptA, initially with its disordered N terminus, and then through binding of kTHB domain 1. (C) Interactions between FptA and TonB1 possibly act to induce movement of the receptor plug domain, allowing for the unstructured N terminus of PyoS5 to thread through the receptor and access the periplasm. Following entry to the periplasm, the N terminus of PyoS5 binds to TonB1 though the TonB-box motif. The formation of the PyoS5-TonB1 complex enables coupling to inner membrane (IM) protein targets of TonB1. (D) This coupling provides energy transduction from the PMF that facilitates the translocation of PyoS5 through the outer membrane. (E) Finally, this results in PyoS5 translocation into the periplasm.

In Fig. 5, we present a unifying model for TonB1-dependent pyocin import based on our data for PyoS5 and that presented by White et al. for PyoS2 (22). CPA binding likely orients the pyocin horizontally with respect to the membrane since the predicted CPA-binding groove in PyoS5 is perpendicular to the long axis of PyoS5. This orientation assumes that CPA molecules are projected vertically from the surface from their LPS anchors. After this initial surface association, we postulate that pyocins use their disordered N terminus to find their transporter, the binding of which causes the pyocin to reorient, allowing the N-terminal kTHB domain to engage the transporter (as found in the PyoS2-FpvAI complex). Similar “fishing pole” models have been proposed for receptor-bound colicins finding translocator proteins, but in these instances, the receptor is generally an outer membrane protein (29). Following opening of the transporter channel by TonB1, the pyocin’s own TonB1 binding site enters the periplasm. A second PMF-dependent step then occurs in which TonB1 in conjunction with the PMF unfolds the kTHB domain of the pyocin and pulls it through the transporter. Whether this energized interaction is responsible for the entire pyocin entering the periplasm (as shown in Fig. 5) or whether domain refolding in the periplasm contributes to the entry process remains to be established.

## MATERIALS AND METHODS

Pyochelin was synthesized as described previously (30). Chromatography columns were purchased from GE Healthcare.

### Strains and plasmids.

All bacteria (Table 1) were cultured in LB (10 g/liter tryptone, 10 g/liter NaCl, 5 g/liter yeast extract [pH 7.2]) at 37°C at 120 rpm shaking, unless otherwise stated. Liquid cultures were inoculated from single colonies on LB agar (1.5% [wt/vol]) plates. M9 medium (8.6 mM NaCl, 18.7 mM NH_4_Cl, 42.3 mM Na_2_HPO_4_, 22.0 mM KH_2_PO_4_) was supplemented with 0.4% (wt/vol) glucose, 2 mM MgSO_4_, and 0.1 mM CaCl_2_.

**TABLE 1:** Bacterial strains used in this study

Strain	Relevant characteristics	Source (reference)
E. coli		
NEB5α	*fhuA2* Δ(*argF-lacZ*)*U169 phoA glnV44* Φ80Δ(*lacZ*)M15 *gyrA96 recA1 relA1 endA1 thi-1 hsdR17*	New England BioLabs
BL21(DE3)	*fhuA2* [*lon*] *ompT gal* (λ DE3) [*dcm*] Δ*hsdS* λ DE3 = λ sBamHIo ΔEcoRI-B *int*::(*lacI*::PlacUV5::T7 gene1) i21 Δ*nin5*	New England BioLabs
TNE012	*ompA ompB tsx*	(50)
P. aeruginosa		
PAO1	Clinical isolate	Manoil lab Washington mutant library
YHP17	Clinical isolate	This study
PAO6609	met-9011 *amiE200 strA pvd-9*	(26)
K1407	PAO6609 *tonB1*	(23, 51)
K1408	PAO6609 *tonB2*	(23)
MS231	PAO6609 *tonB3*	(52)
MS233	PAO6609 *tonB2 tonB3*	(52)
PW8161	PAO1 *fptA*	Manoil lab Washington mutant library
PAO1 Δ*rmd*	CPA deficient	(53)

### Molecular biology.

Genes were amplified from genomic DNA or synthesized by Genewiz. Plasmids were created by restriction enzyme digestion and ligation or whole plasmid mutagenesis. Chemically competent E. coli NEB5α and BL21(DE3) strains were purchased from NEB. Antibiotics were used at the following final concentrations: 100 μg/ml ampicillin and 50 μg/ml kanamycin, and 50 μg/ml gentamicin, all from stock solutions in water; and 37 μg/ml chloramphenicol and 10 μg/ml tetracycline from stock solutions in ethanol.

### Expression and purification of bacteriocins.

PyoS5 and its derivatives, as well as ColB PyoS5, PyoS5-ColIa, PyoS2, PyoS4, and ColIa were expressed heterologously (Table 2) from E. coli BL21(DE3) for 3 h at 37°C or overnight at 20°C while shaking at 120 rpm. For constructs containing the PyoS5 pore-forming domain (amino acid residues 315 to 498), the cells were cotransformed with pHB22, which carries the ImS5 immunity protein, for increased yield. The bacteria were harvested at 5,050 × *g* for 15 min at 10°C, resuspended in binding buffer (0.5 M NaCl, 20 mM Tris-HCl [pH 7.5]), and sonicated on ice. They were then centrifuged at 12,500 × *g* for 20 min at 4°C, filtered through a 0.45-μm syringe filter, loaded onto a 5-ml HisTrap high-performance (HP) column equilibrated in binding buffer, and eluted by gradient elution using elution buffer (binding buffer plus 0.75 M imidazole). The protein was then dialyzed into size exclusion buffer (150 mM NaCl, 20 mM Tris-HCl [pH 7.5]) using a 12- to 14-kDa molecular weight cutoff membrane (Spectra/Por; Spectrum), filtered through a 0.45-μm syringe filter, and applied to a 26/60 Superdex 200 size exclusion chromatography column.

**TABLE 2:** Expression plasmids used in this study

Plasmid name	Protein expressed	Description	Parent vector	Source or reference
pPW18	PyoS5	*PyoS5* with a C-terminal His_6_ tag cloned into the NdeI/XhoI sites	pET21a(+)	This study
pHB18	PyoS5_1–315_	*PyoS5*_1–315_ with a C-terminal His_6_ tag cloned into the NdeI/XhoI sites	pET21a(+)	This study
pHB32	PyoS5_1–315_-Cys	Derivative of pHB18, containing *PyoS5*_1–315_ with a C-terminal cysteine followed by a C-terminal His_6_ tag	pET21a(+)	This study
pHB40	PyoS5_1–315_ Δ2–39	Derivative of pHB18	pET21a(+)	This study
pHB42	PyoS5_1–315_ Δ2–9	Derivative of pHB18	pET21a(+)	This study
pHB41	PyoS5_1–315_ Δ10–13	Derivative of pHB18	pET21a(+)	This study
pHB43	PyoS5_1–315_ Δ16–20	Derivative of pHB18	pET21a(+)	This study
pHB46	PyoS5_1–315_ Δ10–13-Cys	Derivative of pHB32	pET21a(+)	This study
pHB19	PyoS5_1–196_	*PyoS5*_1–196_ with a C-terminal His_6_ tag cloned into the NdeI/XhoI sites	pET21a(+)	This study
pHB33	PyoS5_1–196_-Cys	Derivative of pHB19, containing *PyoS5*_1–196_ with a C-terminal cysteine followed by a C-terminal His_6_ tag	pET21a(+)	This study
pHB24	PyoS5_194–315_	*PyoS5*_194–315_ with a C-terminal His_6_ tag cloned into the NdeI/XhoI sites	pET21a(+)	This study
pHB34	PyoS5_194–315_-Cys	Derivative of pHB24, containing *PyoS5*_194–315_ with a C-terminal cysteine followed by a C-terminal His_6_ tag	pET21a(+)	This study
pHB09	ColB PyoS5	*ColB*_1–340_ translationally fused to *PyoS5*_303–498_ with a C-terminal His_6_ tag cloned into the NdeI/XhoI sites	pET21a(+)	This study
pHB47	PyoS5-ColIa	*PyoS5*_1–315_ translationally fused to *ColIa*_485–626_ with a C-terminal His_6_ tag cloned into the NdeI/XhoI sites	pET21a(+)	This study
pHB22	ImS5	*ImS5* with a stop codon cloned into the NdeI/XhoI sites	pACYCDuet-1	This study
pHB04	FptA	*FptA* with OmpF signal sequence cloned into the NcoI/SacI sites	pBAD/His-MycB	This study
pPW17	TonB1	*TonB1*_109–342_ with N-terminal His_6_ tag followed by TEV cleavage site, cloned into the NcoI/SacI sites	pETM11	(22)
pHB25	TonB-B1 hybrid	E. coli *TonB*_1–102_ translationally fused to P. aeruginosa *TonB1*_201–342_ cloned into the NdeI/XhoI sites	pACYCDuet-1	This study
pNGH131	ColIa	*ColIa* with a C-terminal His_6_ tag cloned into the NdeI/XhoI sites	pET21a	This study
pNGH243	ImS4	*ImS4-His6* cloned into the NcoI*/*HindIII sites	pET24a	This study
pNGH246	PyoS4-ImS4	*PyoS4-ImS4-His6* cloned into the NdeI*/*XhoI sites	pACYCDuet-1	This study
pPW02	PyoS2-ImS2	*PyoS2-ImS2-His6* cloned into the NdeI*/*XhoI sites	pET21a	(22)

PyoS4 was expressed at 28°C in the presence of an additional copy of ImS4(pNGH243) and purified on an S200 16/60 size exclusion column.

Mass spectrometry indicated that all bacteriocins purified without their N-terminal methionines, with the exception of PyoS5_1–315_ Δ2–20, PyoS5_194–315_, and PyoS5_194–315_-Cys.

### Expression and purification of TonB1 soluble fragments.

The TonB1 construct was purified by HisTrap HP column, as described for PyoS5, and then incubated in 300 mM NaCl and 50 mM Tris-HCl (pH 7.0) with 0.07 mg/ml His_6_-tobacco etch virus protease (His_6_-TEV) at room temperature (RT) for 4.5 h. TonB1 was then purified by affinity chromatography on a HisTrap HP column and by size exclusion chromatography on a 26/60 Superdex 200 column.

### Expression and purification of FptA.

FptA purification was modeled after a previous BtuB purification protocol (31). FptA was expressed heterologously from E. coli TNE012 at 37°C while shaking at 120 rpm in LB, and upon reaching an optical density at 600 nm (OD_600_) of 0.6, it was induced with 0.15% (wt/vol) arabinose and supplemented with 0.15% (wt/vol) glucose. The bacteria were harvested as described for PyoS5 and resuspended in 10 mM Tris-HCl (pH 8.0) and 0.25% (wt/vol) lithium diiodosalicylic acid (LIS), sonicated as described for PyoS5, and centrifuged at 4,000 × *g* for 20 min at 4°C. The supernatant was collected, and the pellet was resuspended in fresh buffer and centrifuged again. Both supernatants were ultracentrifuged at 200,000 × *g* for 45 min at 4°C. The pellet was homogenized in 10 mM Tris-HCl (pH 8.0), 0.25% (wt/vol) LIS, and 2% (vol/vol) Triton X-100 and ultracentrifuged again. The resulting pellet was homogenized in 10 mM Tris-HCl (pH 8.0) and ultracentrifuged again. The resulting pellet was homogenized in 10 mM Tris-HCl (pH 8.0) plus 2% (wt/vol) β-OG and 5 mM ethylenediaminetetraacetic acid (EDTA) and ultracentrifuged again. FptA was purified from the supernatant by anion-exchange chromatography. A 5-ml HiTrap DEAE fast flow (FF) column was equilibrated in buffer E (50 mM Tris-HCl [pH 7.5], 1% [wt/vol] β-OG, 5 mM EDTA) and gradient eluted with buffer F (buffer E plus 1 M LiCl). This was followed by 16/60 Sephacryl 300 size exclusion chromatography in buffer E and anion-exchange chromatography on a Mono Q 4.6/100 PE column in buffer E, with gradient elution with buffer F.

### Protein quantification.

All protein concentrations were measured using the absorbance at 280 nm, which was converted to concentration using the sequence-based predicted molar extinction coefficient (ExPASy ProtParam). The presence of scattering impurities, such as protein aggregates, was checked for by measuring the absorbance at 320 nm. All protein masses were confirmed by denaturing electrospray ionization (ESI) mass spectrometry (MS) performed on proteins diluted in formic acid.

### Pyocin cytotoxicity assays.

P. aeruginosa YHP17 cells were grown to an OD_600_ of 0.6, and 200 μl of the culture was mixed with melted, 50°C, soft LB agar (0.75% [wt/vol] agar) and poured over an LB agar plate. Once the plate had set, 2.5 μl of each bacteriocin concentration was spotted onto the plate. The plates were left to dry and then incubated at 37°C overnight.

### LPS-derived polysaccharide isolation.

LPS-derived polysaccharides were isolated as described previously (13). Briefly, 1 liter of cells was grown for 20 h at 37°C, pelleted at 6,000 × *g* for 20 min, and resuspended in 10 ml of 50 mM Tris (pH 7.5), 2 mg/ml lysozyme, and 0.5 mg/ml DNase I. Cells were lysed by sonication, as described for PyoS5 isolation, the lysate was incubated for 30 min at RT, and then 0.2 mM EDTA added. An equal volume of aqueous phenol was then added and the mixture heated for 20 min at 70°C with mixing. The solution was incubated on ice for 30 min and centrifuged at 7,000 × *g* for 20 min, and the aqueous upper layer was extracted. Proteinase K (0.05 mg/ml) was added and the solution dialyzed overnight against 5 liters of distilled water (dH_2_O), followed by dialysis against 5 liters of fresh dH_2_O for 5 h. LPS was pelleted by ultracentrifugation for 1 h at 100,000 × *g* and the pellet resuspended in 10 ml dH_2_O. The suspension was heated at 60°C for 30 min, acetic acid was added, and the mixture was heated at 96°C for 1.5 h. Lipid A was pelleted by centrifugation at 13,500 × *g* for 3 min, and the supernatant, which contains the polysaccharide, was extracted with 10 ml chloroform. The aqueous phase was then lyophilized.

### Biophysical methods.

Native mass spectrometry was performed in 100 mM ammonium acetate buffer, with the exception of TonB1, which was analyzed in 200 mM ammonium acetate buffer.

SPR was performed on a Biacore T200 instrument. A series S sensor chip CM5 (GE Life Sciences) was docked and primed into HBS-OG buffer (25 mM HEPES [pH 7.5], 150 mM NaCl, 1% [wt/vol] β-OG). This buffer was used as a running buffer for all SPR experiments.

For amine coupling using the Amine Coupling kit (GE Healthcare), ligand proteins were desalted into immobilization buffer (25 mM potassium phosphate [pH 7.5], 50 mM NaCl) and diluted 10-fold in 10 mM sodium acetate (pH 5.0; GE Life Sciences).

For thiol coupling using the Thiol Coupling kit (GE Healthcare), ligand proteins were incubated with 10 mM dithiothreitol (DTT) for 2 h and then desalted into immobilization buffer diluted 10-fold in 10 mM sodium acetate (pH 5.0; GE Life Sciences) immediately before immobilization.

Analyte proteins were desalted into HBS-OG buffer before application. The contact time for SPR was set to 120 s, the dissociation time to 600 s, and the flow rate to 30 μl/min. Lower analyte concentrations were applied first.

ITC was performed using a MicroCal iTC200 instrument at 25°C in 0.2 M sodium phosphate buffer (pH 7.5). Proteins in the syringe were at a concentration of 150 μM, and polysaccharides in the cell were at a concentration of 7 mg/ml, which was estimated to be 30 μM based on a molecular weight of 10 kDa and the assumption that CPA constitutes 5% of the LPS polysaccharides. The data were fitted to a one-binding-site model in the MicroCal LLC Origin software. As the CPA concentration is estimated, the observed stoichiometry is unlikely to be correct, while Δ*H*, Δ*S*, and *K_d_* are unaffected by the analyte concentration. Errors reported in the text are standard deviations of the average results from two experiments.

SAXS data were collected at the B21 beamline at Diamond Light Source proteins following in-line size exclusion chromatography on a Superdex 200 column and processed using ScÅtter and ATSAS (32, 33). Guinier approximation analysis and P(r) distributions were determined using ScÅtter. Dummy atoms were fit using multiple parallel runs of DAMMIF (34) and refined using DAMMIN (reference). Bead models were converted to maps using Situs (35) and structures fit into the envelopes using Chimera (36). CRYSOL from the ATSAS suite was used to generate the theoretical curve of the crystal structure and to fit it to the SAXS data.

**(i) Circular dichroism.** Proteins were analyzed at 0.1 mg/ml in 10 mM potassium phosphate buffer (pH 7.5) and 20 mM NaCl using a Jasco J-815 spectropolarimeter. Spectra were measured between 260 nm and 190 nm at a digital integration time of 1 s and a 1-nm bandwidth. Each sample spectrum was measured in quadruplicate and averaged. Molar ellipticity was calculated by subtracting the baseline from sample spectra and dividing by the molecular weight, molar concentration, and pathlength in millimeters. Thermal melting curves for proteins were measured at 222 nm between 20°C and 86°C and 4-parameter sigmoidal melting curves were fit to the equation *f* = *y*_0_ + *a*/(1+*e*^(^*^x^* ^−^ *^x^*^0)/^*^b^*) using non-linear regressions in SigmaPlot to determine the melting temperature (*T_m_*), where *x* is the temperature, *x0* is the *T_m_*, *f* is molar ellipticity, *y*_0_ is the minimal molar ellipticity, and *b* is a fitting parameter.

**(ii) Size exclusion multiangle light scattering.** Proteins were separated in 50 mM Tris (pH 7.5) and 150 mM NaCl using a Superdex 200 10/300 GL column and detected by a Wyatt Dawn HELEOS-II 8-angle light-scattering detector and a Wyatt Optilab rEX refractive index monitor linked to a Shimadzu high-performance liquid chromatography (HPLC) system.

### X-ray crystallography.

Pyocin S5 was concentrated to 16 mg/ml in 25 ml Tris-HCl (pH 7.5) and 150 mM NaCl using a VivaSpin 20 column with a 30-kDa molecular weight cutoff (Sartorius). The crystallization screens Index (Hampton Research) and PACT, JCSG+, and Morpheus (Molecular Dimensions) were used to screen for crystals. Crystals were grown in a vapor diffusion sitting drop setup under JCSG+ screen (Molecular Dimensions) condition C7 (10% [wt/vol] polyethylene glycol 3000 [PEG 3000], 0.1 M sodium acetate, 0.1 M zinc acetate [pH 4.5]) at 18°C. The drops contained 100 nl protein and 100 nl buffer. The cryoprotectant solution was 25% glycerol, 10% (wt/vol) PEG 3000, 0.1 M sodium acetate, and 0.1 M zinc acetate (pH 4.5) for cooling the crystals in liquid nitrogen. Diffraction data were collected at beamline ID30A-3 at the European Synchrotron Radiation Facility (ESRF) at a wavelength of 0.9679 Å using an Eiger detector. We collected 225° of data with 0.15° oscillation. The transmission was 20%, and the exposure time was 0.010 s.

The raw data were analyzed in Dials, revealing a P2_1_ space group and yielding a 98.8% complete set of indexed diffraction spots but no anomalous signal. Molecular replacement was carried out using ColIa residues 450 to 624 in Phaser and yielded electron density for the pore-forming domain of PyoS5. The lack of density for the remainder of the protein indicated that the phases, obtained from ColIa, were not sufficient to build a model for the whole protein.

Improved phases were obtained from anisotropy correction of the same data set using Staraniso in AutoProc (37, 38), which allowed a weak anomalous signal to be detected. The partial model from molecular replacement from Dials and the anomalous data from AutoProc were combined for MR-SAD phasing using Phaser (39). An anomalous substructure containing eight metal ions was identified. Based on the type of metal present under the crystallization conditions, these were assumed to be Zn^2+^. The result was additional, visible helical density beyond the pore-forming domain.

Iterations of model building into the visible helical density in Coot and refinement against the complete Dials data set in Buster version 2.10.3 resulted in a model of PyoS5. The model was optimized in Coot (40), followed by one crystallographic refinement in Buster, and then followed by model optimization in Coot and one refinement in Phenix 1.12 (41). Up to then, the whole model was treated as one TLS group. At this point, four new translation-libration-screw-rotation (TLS) groups were created based on similar B-factors as determined in Phenix, comprising residues 40 to 212, 213 to 338, 339 to 395, and 395 to 505. This increased the *R*_work_ and *R*_free_ upon refinement, indicating that the use of multiple TLS groups made the model worse. The refinement process was therefore continued with the whole model treated as one TLS group.

At the end of the model optimization and refinement, the *R*_work_ was 0.212 and the *R*_free_ was 0.272. MolProbity (42) was used to validate the structure and assess its quality, resulting in a MolProbity score of 1.57. At the end of this validation process, the *R*_work_ was 0.225 and the *R*_free_ was 0.275. Figures of the crystal structure were created using CCP4MG (43) and PyMOL (44).

### Fluorescence microscopy.

**(i) Fluorescent labeling of proteins.** Bacteriocins were fluorescently labeled using maleimide AF488 labels via an engineered C-terminal cysteine. To reduce the cysteine, the protein was mixed in a 1:9 ratio with DTT to yield a concentration of 10 mM DTT and incubated for 2 h at RT. To remove aggregates, the protein was centrifuged at 16,000 × *g* for 1.5 min and the supernatant transferred to a new tube. The supernatant was then applied to a 5-ml HiTrap desalting column and desalted into 25 mM Tris-HCl (pH 7.5), 100 mM NaCl, and 1% (wt/vol) β-OG. The protein concentration was measured, and maleimide AF488 was immediately added in 3-fold excess. The reaction was allowed to proceed for 1 h while mixing by rotary inversion in the dark at RT. Then, the reaction was quenched by adding DTT to a final concentration of 5 mM. The solution was centrifuged and desalted as described before. The absorbance at 280 nm and 494 nm was measured using a V-550 UV-visible spectrophotometer (Jasco). Labeling efficiency was determined as described in the manufacturer’s protocol (Alexa Fluor 488 protein-labeling kit; Thermo Scientific). All fluorescently labeled proteins used for microscopy were labeled with more than 95% efficiency.

**(ii) Fluorescent labeling of bacteria.** Coverslips were cleaned by water bath sonication at 50°C for 15 min in 2% Neutracon (Decon) solution, washed in double-distilled water (ddH_2_O), and air dried.

Bacteria were grown overnight in LB medium. One milliliter of this overnight culture was pelleted, resuspended in 10 ml of supplemented M9 medium, and grown until an OD_600_ of 0.6 was reached. Six hundred microliters of this culture was used per condition. All pelleting steps were performed at 7,000 × *g* for 3 min at RT.

For CCCP treatment, CCCP was added to a final concentration of 100 μM from a 10 mM stock in dimethyl sulfoxide (DMSO) to the bacteria before the addition of the fluorescently labeled protein. The bacteria were incubated with CCCP while mixing by rotary inversion at RT for 5 min, while all other samples were incubated without CCCP for the same time. Fluorescently labeled protein was then added to a concentration of 1 μM and the sample incubated in the dark while mixing by rotary inversion for 20 min at RT.

For trypsin treatment, trypsin was added to a final concentration of 0.1 mg/ml immediately after the incubation with the fluorophore-labeled pyocin. The bacteria were incubated with or without trypsin at 30°C for 1 h at 120 rpm.

Subsequently, bacteria were washed three times in supplemented M9, where each wash consisted of pelleting the bacteria, removing the supernatant, resuspending the pellet in 50 μl by repeated pipetting (10 times) with a P20 pipette, transferring the 50 μl to a new tube with 450 μl of supplemented M9, and vortexing. The bacteria were resuspended in a final volume of 30 μl. Three microliters was applied to an agar pad for microscopic analysis. Agar pads were prepared using Gene Frames (Thermo Scientific) as follows. Supplemented M9 agar (1% [wt/vol]) was prepared and 190 μl pipetted into the Gene Frame. Using a coverslip, the surface was flattened and excess agar removed. Once the agar solidified, the coverslip was removed, the bacterial suspension was added, and a new coverslip was attached to the adhesive side of the Gene Frame.

### Image collection.

All images were collected on an Oxford Nanoimager S microscope at 100-ms exposure. For every image, 200 frames were collected and averaged. Green fluorescence (excitation, 473 nm; emission, 425/50 nm) was measured at 35% laser power.

### Data analysis.

In ImageJ, the 200 collected frames per image were merged using the command “Z project.” Bacterial cells and background were identified in transillumination images using Trainable Weka Classifier. Regions of interest were transferred to green fluorescence images and the mean fluorescence of cells, signal, and background noise quantified. Each image contained a minimum of 15 bacterial cells. For each repeat, a minimum of six images were collected per sample, and three independent experiments were performed for each experiment. As a result, a minimum of 270 bacterial cells were quantified for each sample. Student’s *t* tests were performed to determine *P* values.

### Sequence and structure comparisons.

Sequences were compared using NCBI BLASTn and BLASTp (45), MUSCLE (46), and jackhmmer (47). Similar structures were searched for using NCBI VAST (48) and eFOLD (49).

### Data availability.

The data supporting the findings of the study are available from the corresponding author upon request. The crystallography data from this publication have been deposited to the PDB database (https://www.rcsb.org/) and assigned the identifier 6THK.
