# Supplementary figures and images for: Pyocin S5 Import into Pseudomonas aeruginosa Reveals a Generic Mode of Bacteriocin Transport
Source: mBio. 2020 Mar 10;11(2):e03230-19. doi: 10.1128/mBio.03230-19 (PMC7064778; doi:10.1128/mBio.03230-19)

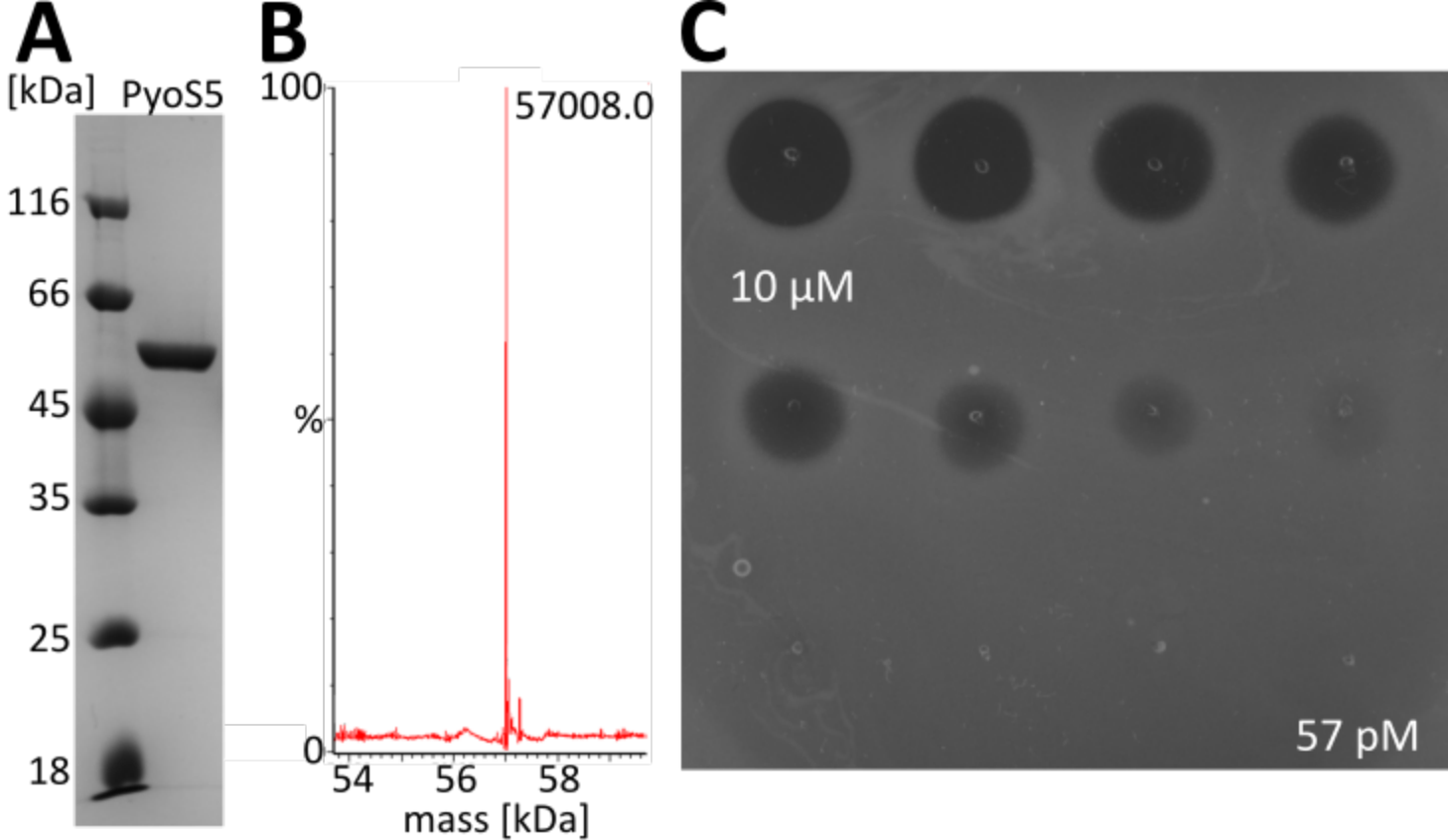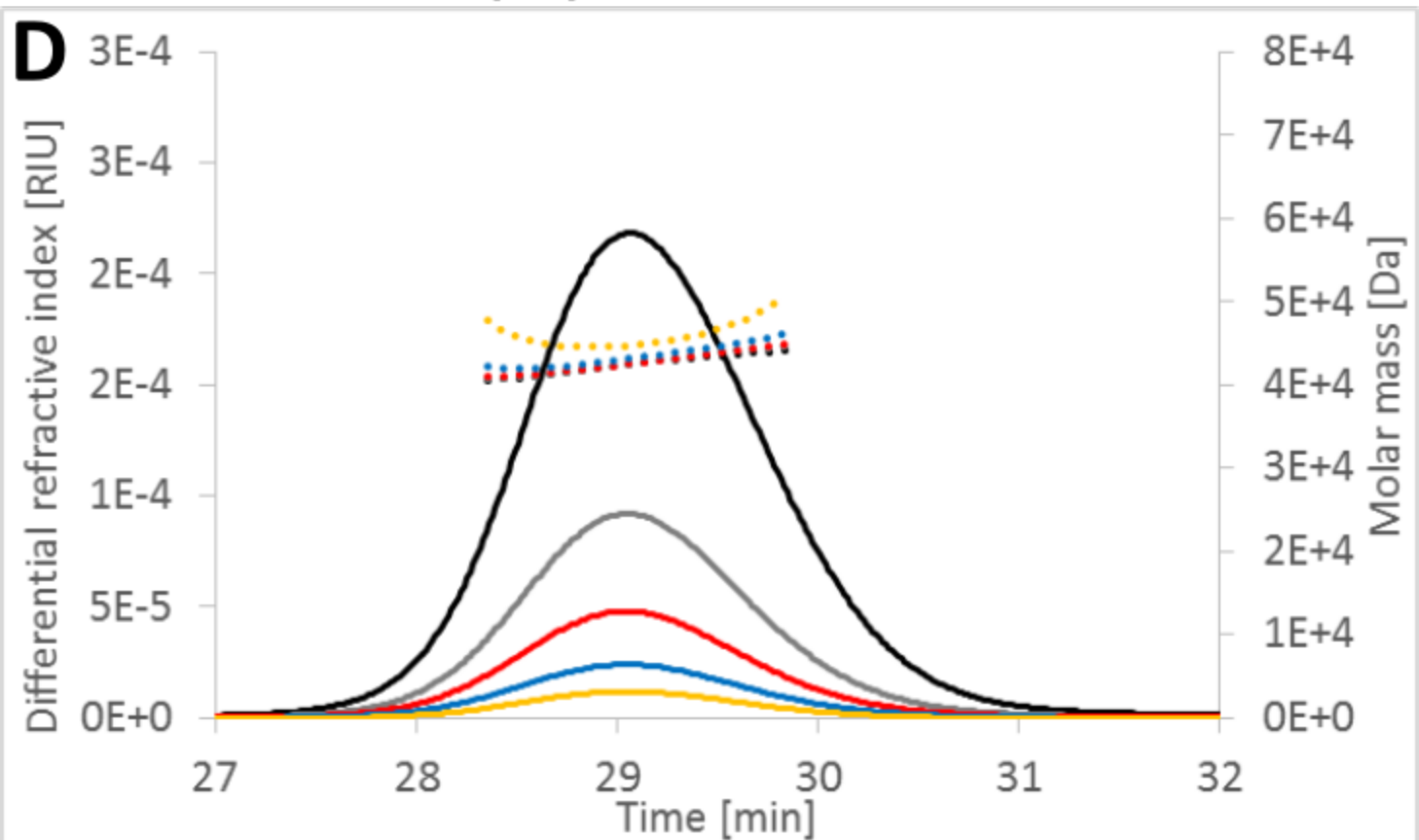

Supplement: FIG S1 [file mBio.03230-19-sf001.pdf]

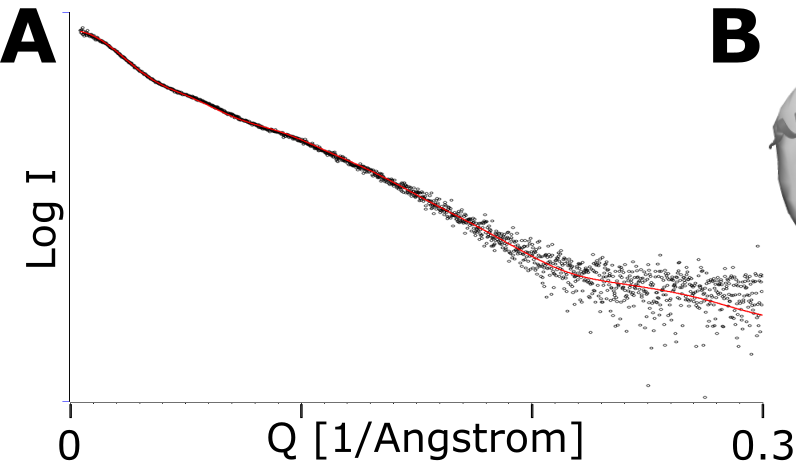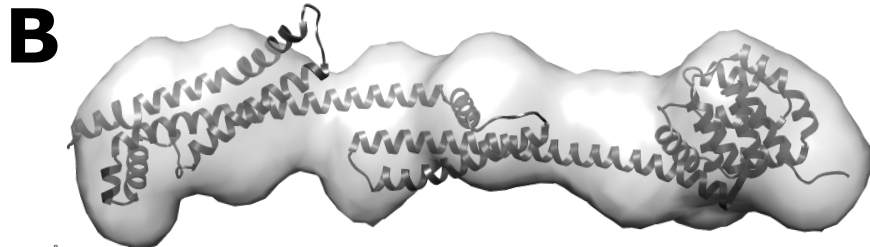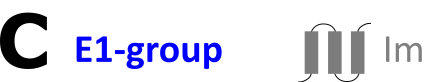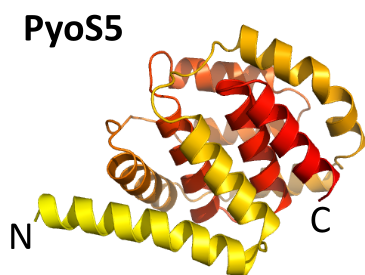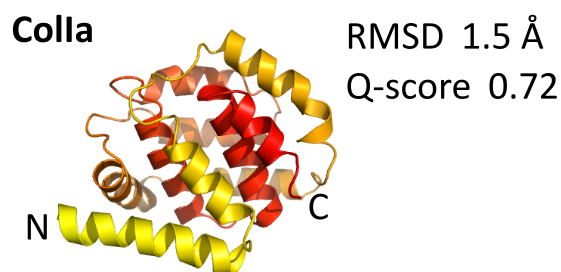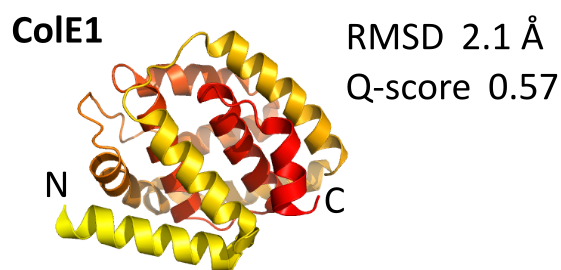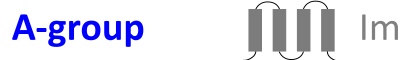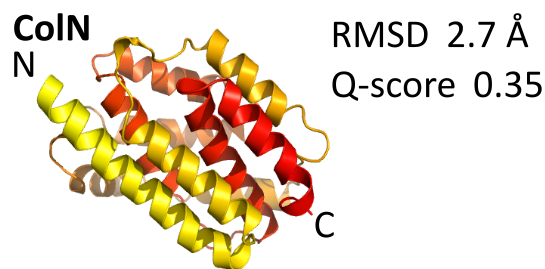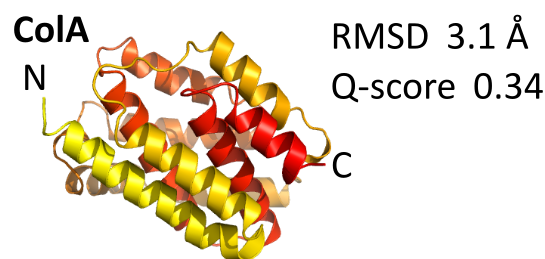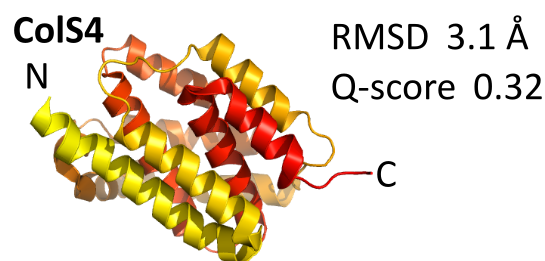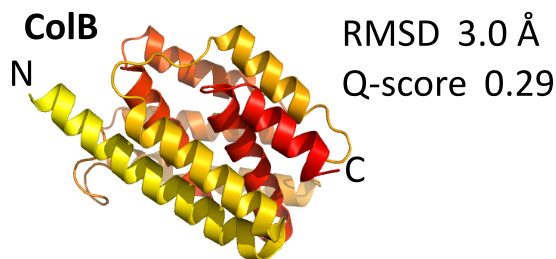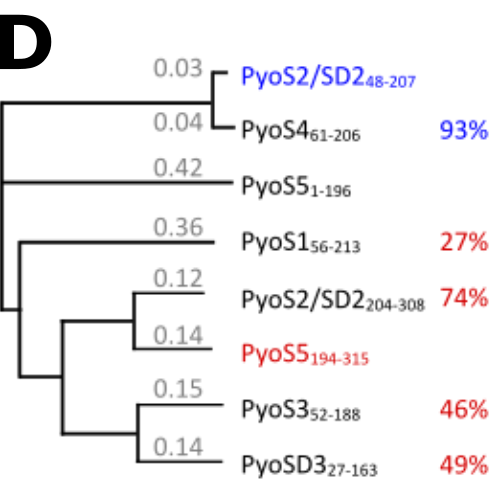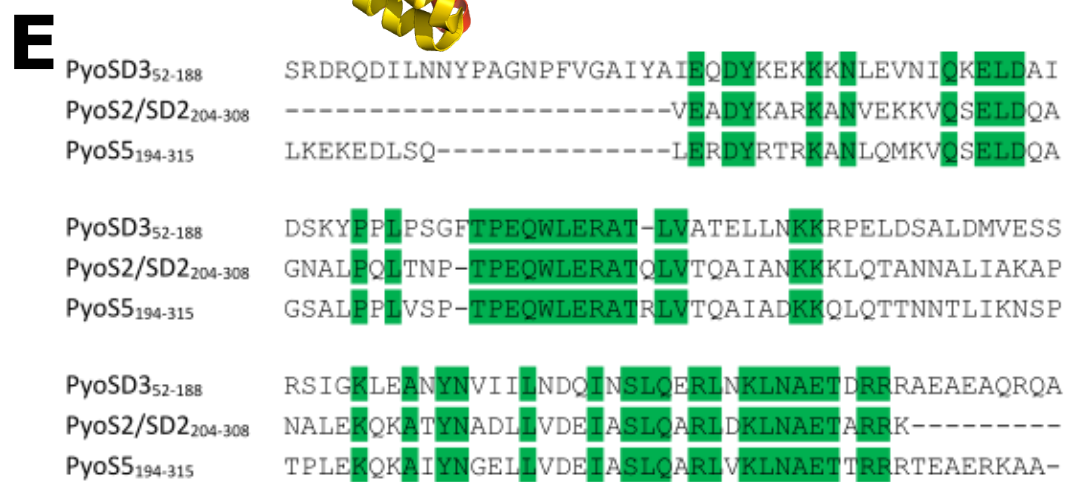

Supplement: FIG S2 [file mBio.03230-19-sf002.pdf]

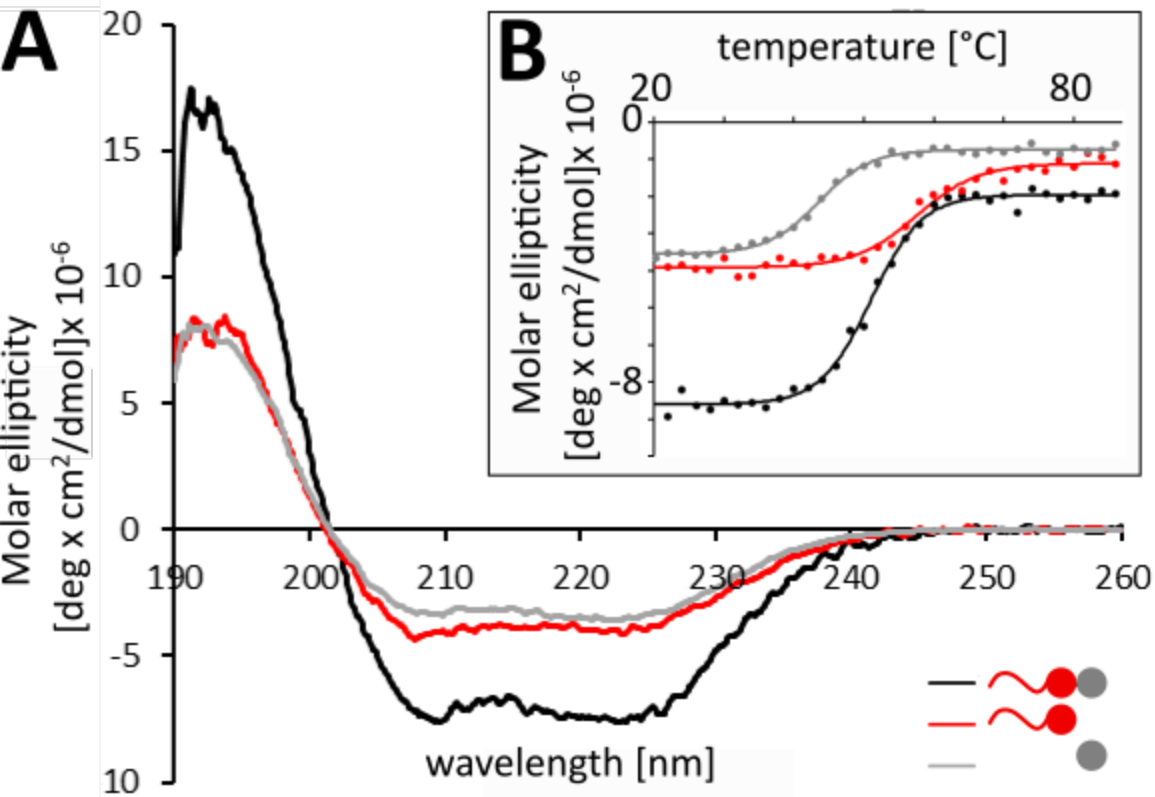

Supplement: FIG S3 [file mBio.03230-19-sf003.pdf]

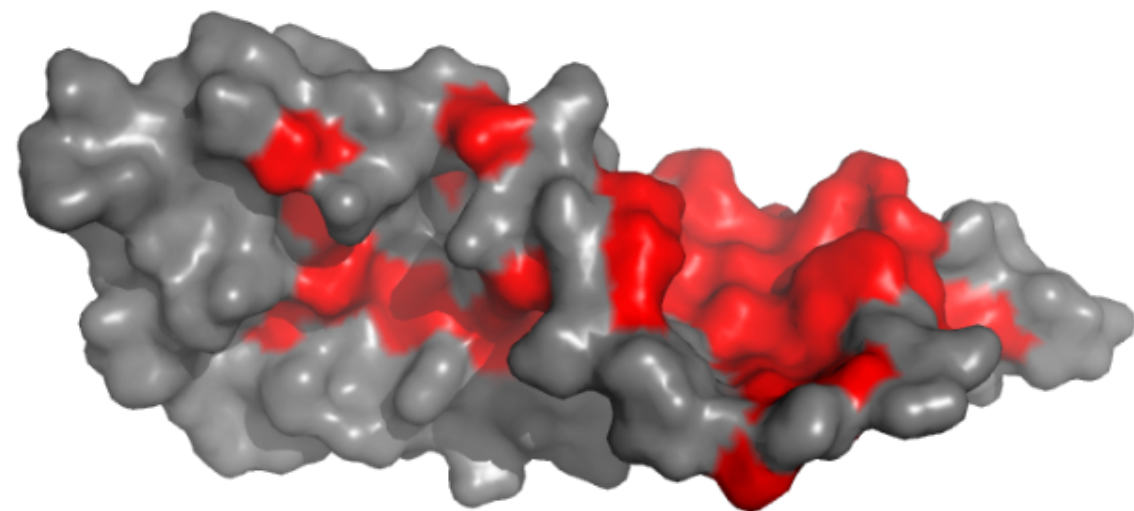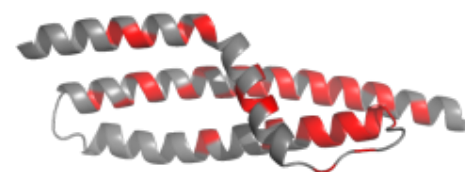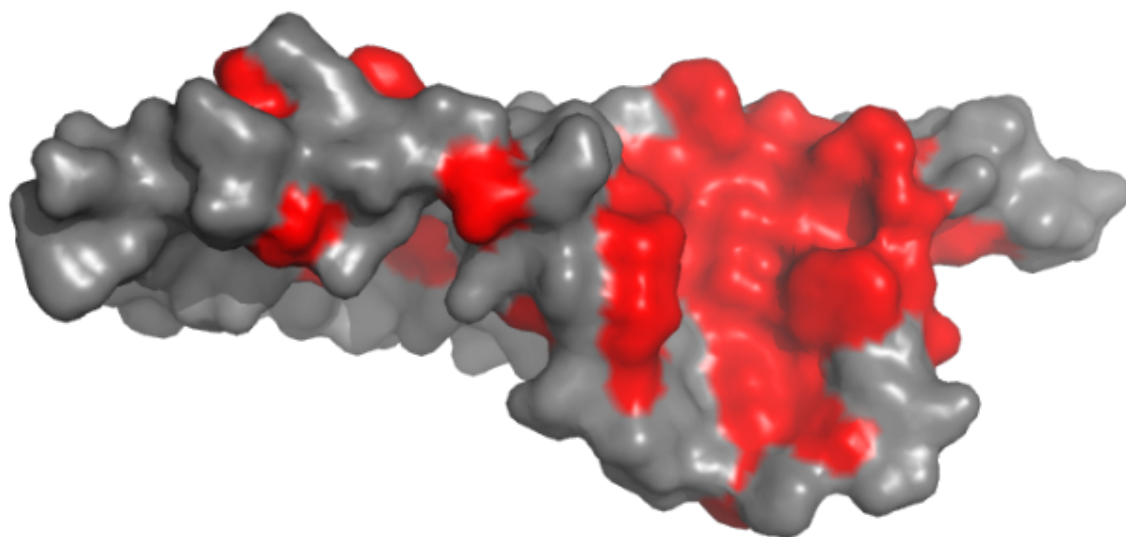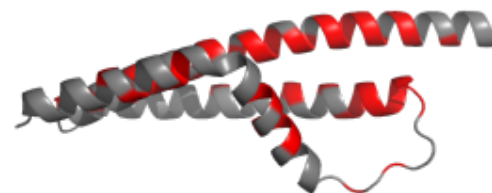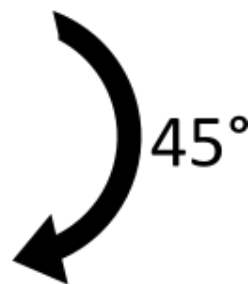

Supplement: FIG S4 [file mBio.03230-19-sf004.pdf]

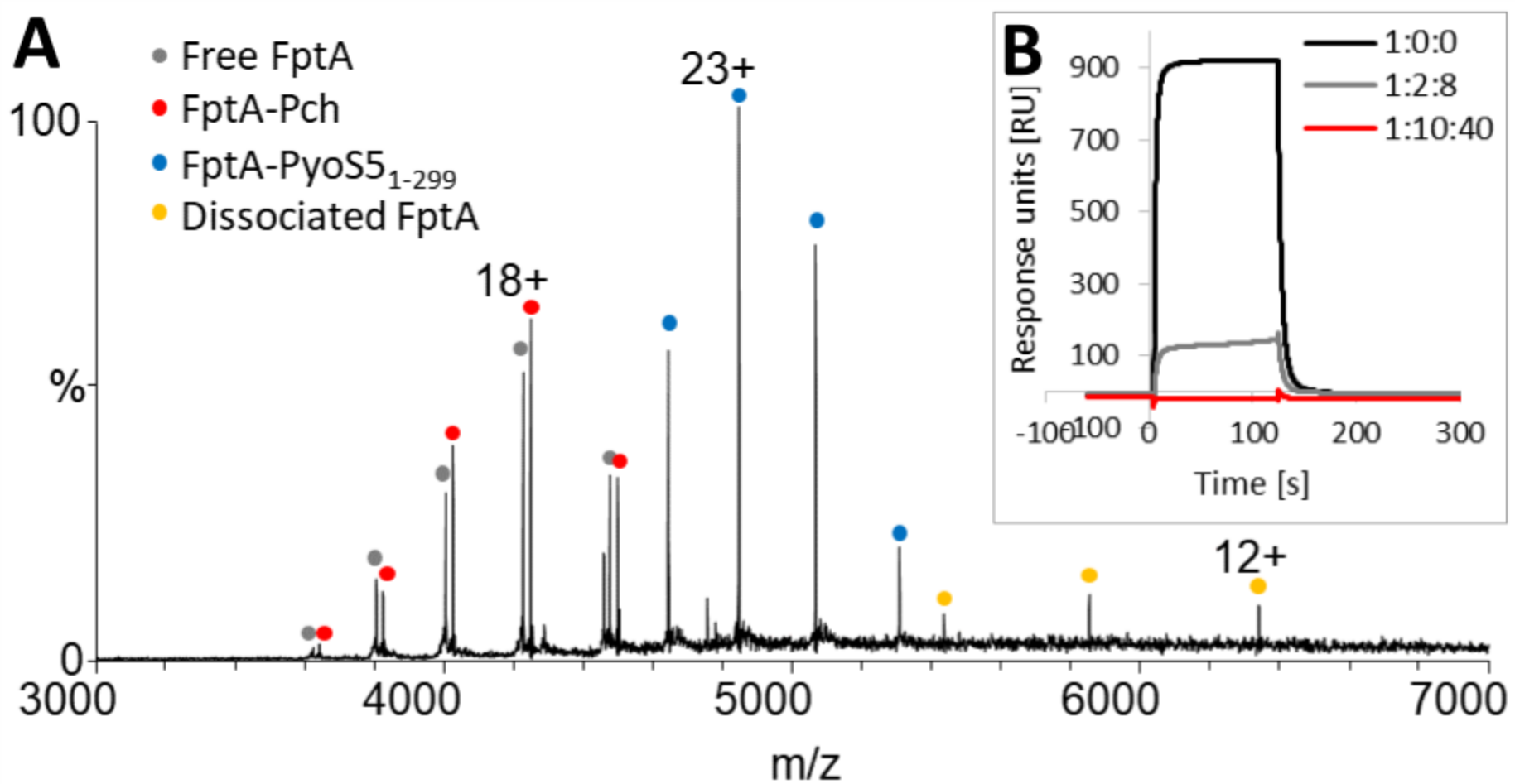

Supplement: FIG S5 [file mBio.03230-19-sf005.pdf]

**A**

PA06609 (parent)

K1408 (TonB2<sup>-</sup>)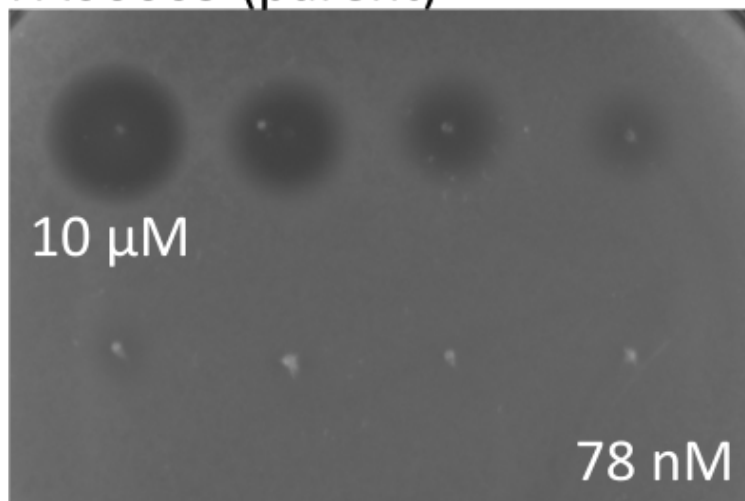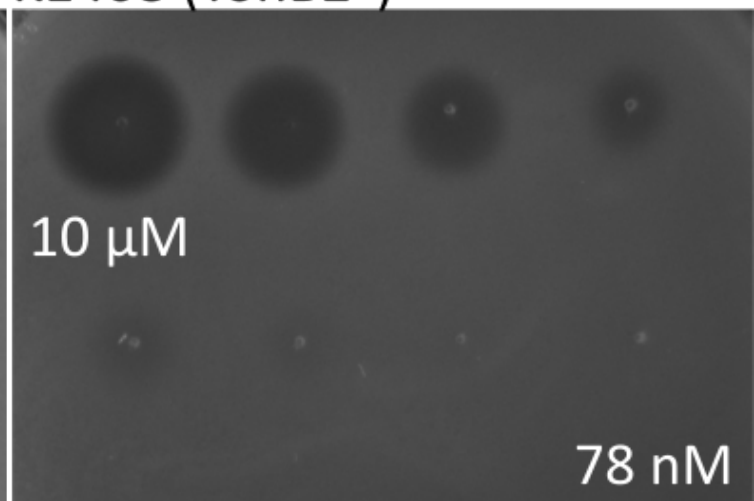MS231 (TonB3<sup>-</sup>)MS233 (TonB2<sup>-</sup> TonB3<sup>-</sup>)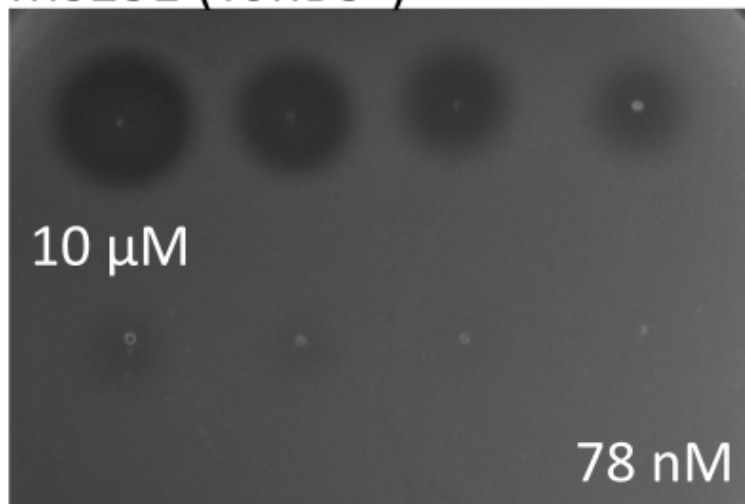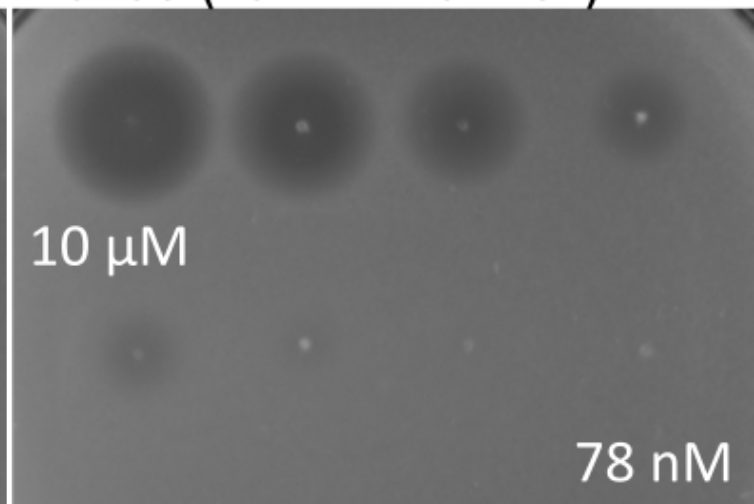**B**

PAO1

 $\Delta$ rmd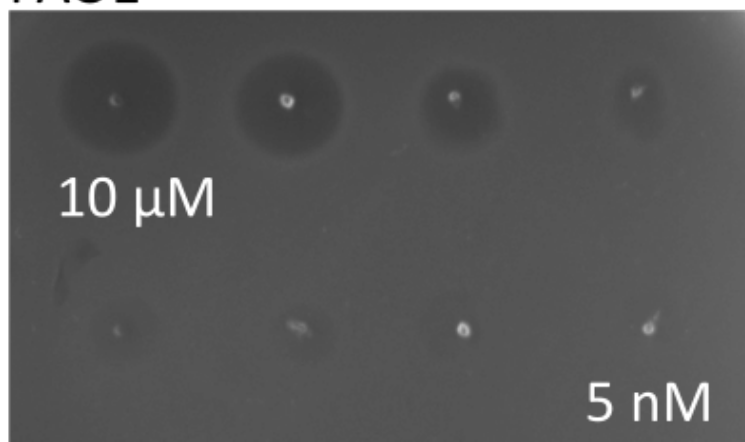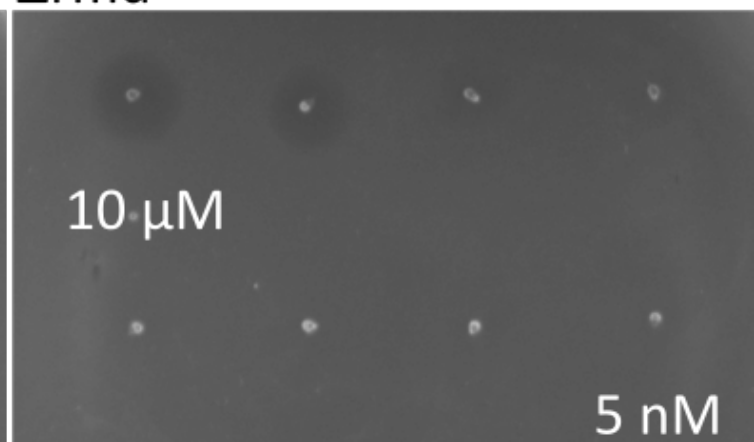

Supplement: FIG S6 [file mBio.03230-19-sf006.pdf]

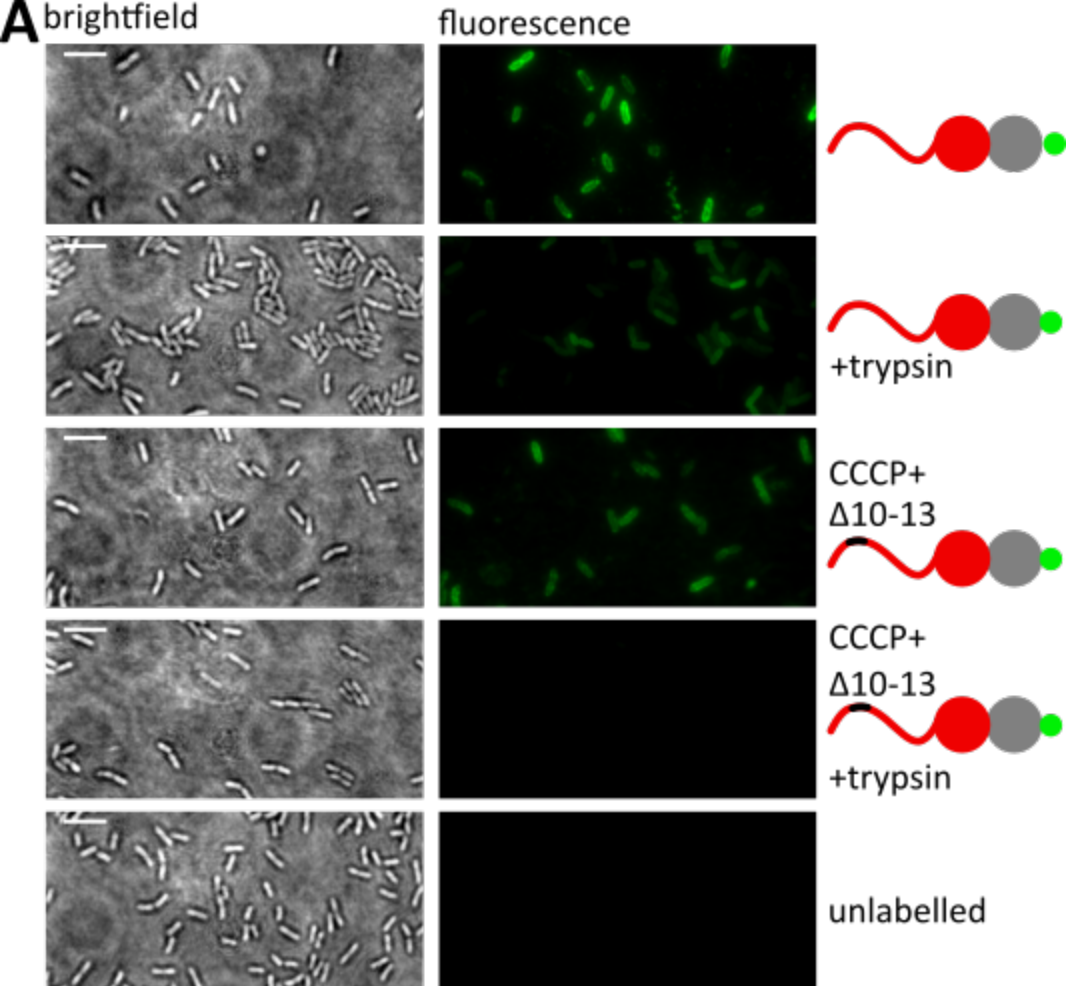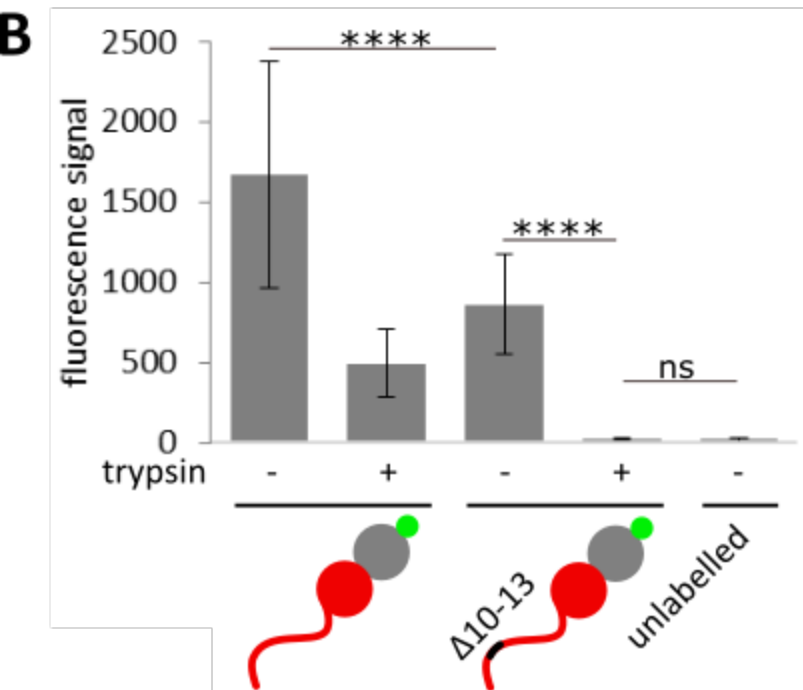

Supplement: FIG S7 [file mBio.03230-19-sf007.pdf]

| PyoS2     | PyoS4     | PyoS5     | ColBPyoS5 | PyoS2     | PyoS4     | PyoS5     | ColBPyoS5 |
|-----------|-----------|-----------|-----------|-----------|-----------|-----------|-----------|
| 1 $\mu$ M |

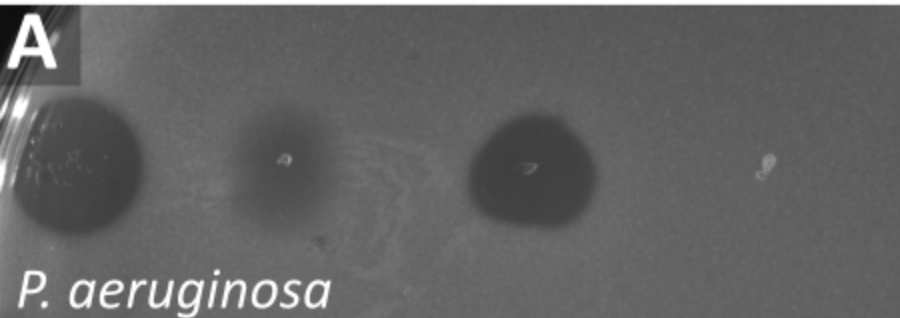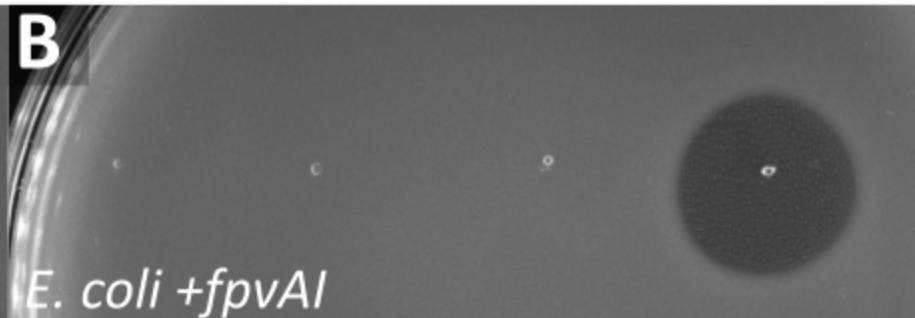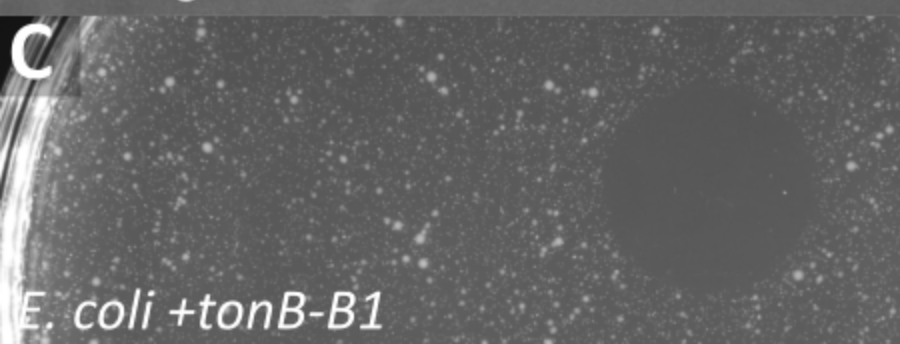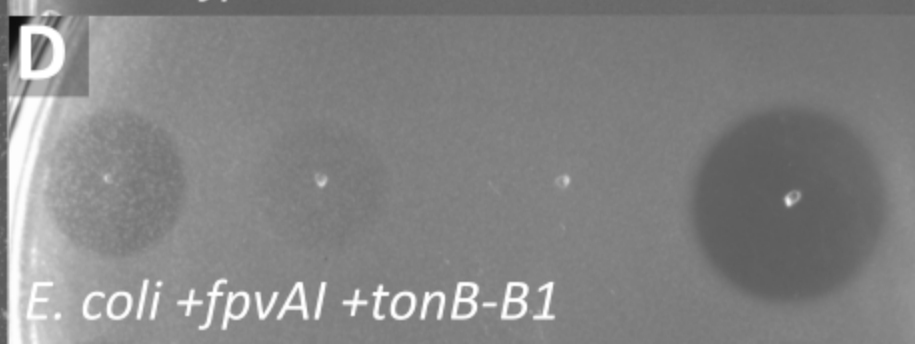

Supplement: FIG S8 [file mBio.03230-19-sf008.pdf]
