# Supplementary material for: Pyocin S5 Import into Pseudomonas aeruginosa Reveals a Generic Mode of Bacteriocin Transport
Source: mBio. 2020 Mar 10;11(2):e03230-19. doi: 10.1128/mBio.03230-19 (PMC7064778; doi:10.1128/mBio.03230-19)
Supplement: TABLE S1 [file mBio.03230-19-st001.docx]

Supplementary Table S1: X-ray data processing, refinement and validation statistics for PyoS5.

| **Data Processing** | |
| --- | --- |
| X-ray wavelength | 0.9679 Å |
| Space group | P 1 2_1_ 1 |
| Cell dimensions | a = 50.246 Å, b = 52.878 Å, c = 104.807 Å |
| Cell angles | α = 90°, β = 95.16°, γ = 90° |
| Resolution | 53.88-2.20 Å (2.27-2.20 Å) |
| Unique reflections | 28285 (2473) |
| Completeness | 98.8 % (99.8 %) |
| Multiplicity | 4.4 (4.6) |
| CC(1/2) | 0.996 (0.728) |
| R_meas_ | 0.179 (2.52) |
| **Refinement** |  |
| R_work_/R_free_ | 0.225/0.275 |
| Number of protein atoms | 3633 |
| Number of Zn^2+^ atoms | 8 |
| Average B-factor | 63.91 Å^2^ |
| **Validation** |  |
| RMS bonds | 0.0096 Å |
| RMS angles | 1.05° |
| Ramachandran favoured | 98.49% |
| Ramachandran allowed | 1.51% |
| Ramachandran outliers | 0% |
| MolProbility Clashscore | 2.76 |

Values in parenthesis denote highest resolution shell.
