# Supplementary material for: Pyocin S5 Import into Pseudomonas aeruginosa Reveals a Generic Mode of Bacteriocin Transport
Source: mBio. 2020 Mar 10;11(2):e03230-19. doi: 10.1128/mBio.03230-19 (PMC7064778; doi:10.1128/mBio.03230-19)
Supplement: TABLE S2 [file mBio.03230-19-st002.docx]

**Supplementary Table S2: Interactions of PyoS5 with cell envelope components.**

| **A** | **K_d_s of PyoS5 constructs binding to LPS-derived polysaccharides by ITC** | | | | | |
| --- | --- | --- | --- | --- | --- | --- |
| Polysaccharide in the cell at 7 mg/mL | | Titrant with concentration | K_d_ [nM] | ΔH [kcal/mol] | ΔS [cal/mol/K] | N |
| *P. aeruginosa* PAO1 LPS-derived polysaccharide (CPA and OSA) | | 150 μM PyoS5_1-315_ | 612 ±332 | -18 ±6 | -32 ±22 | 0.16 ±0.04 |
| *P. aeruginosa* PAO1 LPS-derived polysaccharide (CPA and OSA) | | 150 μM PyoS5_1-196_ | No binding was observed |  |  |  |
| *P. aeruginosa* PAO1 LPS-derived polysaccharide (CPA and OSA) | | 150 μM PyoS5_194-315_ | 269 ±44 | -14 ±0.3 | -16 ±1 | 0.56 ±0.36 |
| *P. aeruginosa* Δrmd LPS-derived polysaccharide (OSA only) | | 150 μM PyoS5_194-315_ | No binding was observed |  |  |  |
|  | | | | | | |
| **B** | **Complex formation of FptA with PyoS5 constructs observed by native MS** | | | | | |
| Construct(s) | | μM | Expected mass of complex (Da) | | Observed mass (Da) | |
| FptA | | 10 | 76068.27 | | 76066 | |
| FptA + PyoS5_1-315_ | | 10 + 17.3 | 113261.40 | | 113259 | |
| FptA + PyoS5_1-194_ | | 5 + 7.5 | 99797.97 | | 99796 | |
| FptA + PyoS5_194-315_ | | 10 + 7 | 91116.37 | | 76066 | |
|  | | | | | | |
| **C** | **K_d_s determined by SPR at 25 °C, average of three repeats** | | | | | |
| Ligand immobilized by amine coupling | | Amount immobilized [RUs] | Analyte in HBS-OG buffer | | K_d_ | |
| PyoS5_1-315_ | | 3464 | FptA | | 6.5 ±0.4 μM | |
| PyoS5_1-196_ | | 4153 | FptA | | 7.1 ±0.7 μM | |
| PyoS5_194-315_ | | 2088 | FptA | | No binding | |
| PyoS5_1-315_ Δ2-39 | | 9223 | FptA | | 14.7 ±0.4 μM | |
| PyoS5_1-315_ | | 3464 | TonB1 | | 241 ±9 nM | |
| PyoS5_1-196_ | | 4153 | TonB1 | | 231 ±2 nM | |
| PyoS5_194-315_ | | 2088 | TonB1 | | No binding | |
| PyoS5_1-315_ Δ10-13 | | 5324 | TonB1 | | No binding | |
| PyoS5_1-315_ Δ2-9 | | 5352 | TonB1 | | 1.57 ±0.09 µM | |
| PyoS5_1-315_ Δ16-20 | | 7727 | TonB1 | | 3.12 ±0.05 μM | |
| PyoS5_1-315_ Δ2-9 | | 5324 | FptA | | 6.53 ±0.13 µM | |
| PyoS5_1-315_ Δ10-13 | | 5352 | FptA | | 10.07 ±0.19 µM | |
| PyoS5_1-315_ Δ16-30 | | 7727 | FptA | | 36.2 ±4.9 µM | |
